# FTO and MC4R polymorphisms, and selected pre-, peri- and postnatal factors as determinants of body mass index and fatness in children: a thorough analysis of the associations

**DOI:** 10.1186/s40101-023-00344-1

**Published:** 2023-12-08

**Authors:** Ewa Bryl, Paula Szcześniewska, Agata Dutkiewicz, Agnieszka Słopień, Monika Dmitrzak-Węglarz, Tomasz Hanć

**Affiliations:** 1 Institute of Human Biology and Evolution, Faculty of Biology, Adam Mickiewicz University, 61-614 Poznan, Poland; 2 Department of Child and Adolescent Psychiatry, Poznan University of Medical Sciences, 60-572 Poznan, Poland; 3 Psychiatric Genetics Unit, Department of Psychiatry, Poznan University of Medical Sciences, 60-806 Poznan, Poland

**Keywords:** Body composition, Children, BMI, FTO rs9939609, MC4R rs17782313, Environmental factors, ACE

## Abstract

**Background:**

Overweight and obesity among children have become significant global health concerns. Previous studies have highlighted the potential role of genetic factors, particularly polymorphisms in the FTO and MC4R genes, as well as environmental factors in the development of childhood obesity. This study aimed to investigate the relationships between genetic, socioeconomic and perinatal factors, adverse childhood events (ACEs), and lifestyle, and their impact on overweight, obesity and body composition parameters in children. Additionally, we explored potential interactions between genetic factors and ACEs.

**Methods:**

Four hundred fifty-six children aged 6–12 years participated in our study. Information on the socioeconomic status, perinatal factors, ACEs and lifestyle of the children was collected with a questionnaire completed by their parents/guardians. We examined the children’s body weight and conducted an electrical bioimpedance analysis. Overweight and obesity were diagnosed based on the International Obesity Task Force and McCarthy criteria. We genotyped two selected polymorphisms in the FTO and MC4R genes using the TaqMan SNP allelic discrimination method.

**Results:**

Higher BMI (Body Mass Index) z scores were related to higher paternal BMI and lower maternal age at the child’s birth. Higher FMI (Fat Mass Index) z scores were associated with higher paternal BMI, increased gestational weight, lower maternal education and the presence of the FTO risk allele. Higher FatM (fat mass in kg) z scores were linked to lower maternal education, lower maternal age at the child’s birth, higher maternal body weight gain, paternal BMI and the presence of the FTO risk allele. Moreover, interaction effects were observed on BMI z scores between ACE and FTO AA, and on FMI z scores and FatM z scored between ACE and MC4R CC.

**Conclusions:**

The contribution of environmental factors is more strongly related to changes in body composition than genetic ones. Additionally, the presence of the risk allele combined with unfavourable environmental factors like ACEs leads to visible interaction effects, resulting in increased BMI z scores and FMI z scores in children.

**Supplementary Information:**

The online version contains supplementary material available at 10.1186/s40101-023-00344-1.

## Introduction

The problem of overweight and obesity affects an increasing number of people, including children. According to the latest data from the World Health Organization (WHO), 39 million children under the age of 5 were overweight or obese [1]. Research is ongoing to identify factors influencing this phenomenon, which can be categorised as genetic and environmental ones. Genetic factors include gene polymorphisms that may be associated with a higher risk of developing overweight and obesity. The previous meta-analysis indicated that the polymorphism of the FTO rs9939609 gene is related to body weight, BMI (Body Mass Index) and body fat content not only in adults but also in children [2]. In a group of children and adolescents aged 6–19 years, the presence of at least one risk allele of the FTO gene was associated with higher BMI, BMI z scores and adiposity [3, 4]. In the children, the presence of the unfavourable allele nearly doubled the risk of binge eating [3]. Similar findings emerged concerning the MC4R gene polymorphism. The results of studies carried out by Ho-Urriola et al. on a cohort of children also revealed that the MC4R rs17782313 polymorphism may be associated with the risk of overweight and increased food consumption in children aged 6–12 years. The presence of the C allele was associated with increased eating pleasure, reduced satiety and a greater tendency to eat without hunger, which may contribute to childhood obesity [5].

Family environment is a major factor for the children’s health. Children raised by a single parent have a higher risk of experiencing overweight and obesity [6]. In children older than 7, the association between the family structure and the child’s BMI increases and is mediated by household income levels [7]. Low income itself is a risk factor for higher BMI among children [8]. Various parental factors hold importance in shaping children’s BMI and nutritional status. For instance, the mother’s BMI both before and after the child’s birth, the father’s BMI, and the parents’ level of education significantly contribute to this aspect [9, 10]. The children’s BMI values were more frequently within the recommended range for their age when their parents had a higher level of education and a higher income per family member [9, 10]. In Serbia, for instance, mothers’ educational attainment proved to be the most influential factor in determining the children's nu’ritional status [11]. Additionally, there is a positive correlation between parental BMI and the BMI of their children, indicating that higher parental BMI is linked to increased BMI in the children [12]. Specifically, a higher paternal BMI has been identified as a factor contribu’ing to elevated body weight and body fat percentage in the children [13], while a higher maternal BMI before pregnancy increases the risk of overweight and obesity in the offspring [14]. The maternal age also exerts an impact on the children’s body weight. A study conducted by Potocka et al. found that younger maternal age was associated with higher BMI z scores and a higher percentage of energy intake in the children’s diets [15].

Perinatal factors represent additional variables that can have implications for children’s weight and body composition. Both a low (< 2500 g) and high (> 4000 g) birth weight have been associated with potential adverse effects on children’s future health. A low birth weight may be associated with a higher risk of metabolic syndrome and central obesity [16]. On the other hand, a high birth weight may also contribute to a higher risk of overweight and abdominal obesity among children in Poland [17]. Another factor related to the child’s health is breastfeeding. The results of a study conducted in 12 countries show that breastfeeding may protect from obesity and excessive body fat levels in children aged 9–11 years [18]. It is an important component of reducing BMI in children and lowering the risk of obesity [14]. The type of birth also matters. A recent meta-analysis indicates that a c-section (caesarean section) is associated with childhood obesity, yet not overweight [19].

In addition to genetic, socioeconomic and perinatal factors, lifestyle plays an important role in weight changes among children. A healthy diet, regular meals [20, 21], physical activity [21] and limited screen time are protective factors against the development of overweight and obesity in children. Spending more than 2 h [22] or more than 4 h [23] in front of the screen was associated with greater likelihood of overweight/obesity. The length of sleep may also be related to the children's weight. In a study by Resiak et al., sleeping less than 5 h was associated with a higher risk of overweight/obesity in the children [9]. The sleep length is positively affected by a bedtime routine, which is less often observed in households with many children and among the children of mothers with a low education [24], highlighting how all these factors relate.

Previous studies have shown that adverse life events in childhood (ACEs) may have a significant role in changes in the weight and body composition of children [25–29]. Most studies focused on the effect of ACEs on the occurrence of changes in body weight in adulthood [30]. However, there are still no clear conclusions about the impact of ACEs on changes in body weight in childhood. Meta-analyses have shown that ACEs are associated with both childhood obesity [25–27] and underweight [31, 32]. Even less is known about the relationship between ACEs and body composition parameters. A study by Derks et al. conducted among Dutch children shows that ACEs do not have a significant effect on the body composition, and the most important factors are the socioeconomic conditions [28]. However, a study by Deng et al. finds that ACEs are associated with a greater increase in BMI and FMI (Fat Mass Index) in children aged 5–17 years with significant gender differences [29].

The aim of our study was to investigate the potential associations between the BMI z scores, FMI z scores, FFMI (Fat Free Mass Index) z scores, FatM (Fat Mass in kg) z scores and the socioeconomic status, perinatal factors, parental factors, the children’s lifestyle, ACEs and FTO rs9939609 and MC4R rs17782313 polymorphisms of Polish children aged 6–12 years. The study was exploratory.

## Material and methods

The size of the study group was determined based on data published by the Statistics Poland [33], which indicated an approximate population of 36,000 primary school children in Poznan at the time of the research. To calculate the specified confidence level of 95%, a structure index of 18%, and an estimation error of 5%, a sample size of 225 or greater was calculated to ensure a robust representation. The study group consisted of 456 children with a comparable number of boys and girls (52.19 and 47.81%, respectively). The children were aged 6 to 12 years (mean = 8.99, SD = 1.32). The study was conducted between March 2017 and November 2019 across 11 randomly selected state primary schools in Poznan. It was funded by the National Science Centre, Poland, grant number: 2016/21/B/NZ5/00492 and approved by the Institutional Bioethics Board of Poznan University of Medical Sciences (approval no. 542/14). Prior to participation, the parents or legal guardians of the participants received written information about the study along with consent cards and questionnaires.

The collected material included information and measurements of 530 individuals (276 boys and 254 girls) aged 6–12 years (mean = 8.99 SD = 1.32). After eliminating cases with missing information on body composition parameters or the FTO and MC4R gene polymorphisms determination, the final database included 456 children. The data on the socioeconomic status, ACEs and gene polymorphisms were collected for each child, however, there was a small rate of missing data for certain variables, such as family type (*n* = 12, 2.63%), place of residence (*n* = 27, 5.92%), parental subjective assessment of economic situation of the family (*n* = 17, 3.73%), gaining weight during pregnancy (*n* = 20, 4.39%), pregnancy duration (*n* = 31, 6.80%), single/twin delivery (*n* = 19, 4.17%), type of delivery (*n* = 18, 3.95%), birthweight (*n* = 13, 2.85%), breastfeeding (*n* = 13, 2.85%), maternal BMI category before pregnancy (*n* = 28, 6.14%), maternal current BMI category (*n* = 23, 5.04%), maternal educational level (*n* = 15, 3.29%), paternal current BMI category (*n* = 27, 10.31%), paternal educational level (*n* = 32, 7.02%), siblings (*n* = 20, 4.39%), life threat (*n* = 28, 6.14%), witness of life threat (*n* = 28, 6.14%), violence victim (*n* = 28, 6.14%), violence witness (*n* = 30, 6.58%), death of someone close (*n* = 28, 6.14%), family conflicts (*n* = 29, 6.36%), separation from parents (*n* = 30, 6.58%), school problems (*n* = 3, 6.80%), other ACEs (*n* = 33, 7.24%).

### Anthropometric measurements

Each child was measured by qualified medical personnel. Body height was checked with a Seca 213 stadiometer (with an accuracy of 1 mm), and body weight (with an accuracy of 0.01 kg) and electrical bioimpedance with the Tanita MC-780 body composition analyser. During the body composition examination the children wearing light clothing (a blouse or shirt and skirt or trousers), with no outer clothing (a jacket or coat). They were barefoot and placed appropriately on the electrodes while ensuring proper electrode positioning. The values of raw indicators, such as FatM in kg, were analyzed. Subsequently, BMI, FMI, and FFMI were calculated using the following equations: $BMI=\frac{body\;weight\;\left[kg\right]}{{(body\;height[m])}^{2}}$, $FMI=\frac{body\;fat\;mass\;\left[kg\right]}{{(body\;height[m])}^{2}}$, $FFMI=\frac{fat\;free\;body\;mass\;\left[kg\right]}{{(body\;height[m])}^{2}}$.

The BMI was adjusted for sex and age on the basis of WHO growth charts [34] with the use of WHO AnthroPlus software and presented as z scores. FMI, FFMI and FatM were standardised by age and sex within the sample. The diagnosis of overweight, obesity, and underweight was determined following the guidelines of the International Obesity Task Force (IOTF) [35, 36] and based on body fat cut-off points obtained using the bioimpedance method (McCarthy criteria) [37].

### Socioeconomic and lifestyle factors

In the parental questionnaire, questions pertaining to the socioeconomic status enabled the classification of families into three types: both biological parents, a biological parent with a partner, or a single parent. The place of residence was determined based on the number of residents and categorised as a village (< 10 000 residents), small and medium-sized town (10 000 – 100 000 residents) or large city (> 100 000 residents) [38]. According to Gross Domestic Product (GDP) per capita data, the financial situation in the region was comparable across all places of residency [39]. The economic situation was assessed subjectively by the parent and categorised as: bad, average or good. The ‘bad economic situation’ group comprised the children whose parents reported facing serious difficulty or difficulty in meeting the family's needs, while the ‘average economic situation’ group included children whose parents reported some difficulty. The ‘good economic situation’ group consisted of the children whose parents indicated that meeting the family's needs was quite easy or easy. The maternal weight gain during pregnancy was assessed based on the gynaecologist’s opinion and categorised as: ‘exceeded’ or ‘non-exceeded’ in accordance with the guidelines from the Institute of Medicine. The recommended amount of weight gain depends on the pre-pregnancy BMI (for women who had a BMI < 18.5 before pregnancy, the recommended weight gain is 12.5–18 kg; for women with a pre-pregnancy BMI of 18.5–24.9 the recommended weight gain is 11.5–16; women with pre-pregnancy BMI 25–29.9 should aim for weight gain between 7–11.5, and women with pre-pregnancy BMI ≥ 30 should not exceed weight gain between the range of 5–7 kg) [40]. Pregnancy duration was divided into three categories:: < 37 weeks (preterm birth), 37–42 weeks (term birth) and > 42 weeks (post-term birth) [41]. The information on maternal health conditions before and during pregnancy, such as hypertension, diabetes, thyroid diseases, kidney diseases and heart failure was collected from parental responses (yes/no). We distinguished a single or twin pregnancy and a vaginal or c-section delivery. The birthweight was classified into 3 categories: < 2500 g, 2500-4000 g, > 4000 g [42]. Additionally, information on breastfeeding (yes/no) was obtained.

The parents declared their weight and height, on the basis of which we calculated BMI. Maternal BMI before pregnancy, maternal current BMI and paternal current BMI were classified according to WHO guidelines as: underweight, proper weight, overweight or obesity [43]. The educational level of the parents was categorised as: primary (8 years of education), vocational (10 years), secondary (12 years) or university qualifications (bachelor’s – 15 years or master’s degree – 17 years). We asked about the presence of siblings in the household (yes/no). Regarding the children’s lifestyle, sleep length was categorised as ≥ 9 h or < 9 h [44], eating behaviours were classified as either at least 3 regular meals/day + a snack or irregular meals. Physical activity was divided into exercise 3 days/week for ≥ 3 h or less [45], and screen time as ≤ 2 h or more than 2 h [46].

### Measurements of adverse life events in childhood

Measurements of ACEs in the children were collected using a survey method. The parents or legal guardians received forms containing questions related to the occurrence of specific experiences in the child's life. The questionnaire was developed based on selected questions from the Traumatic Events Screening Inventory (TESI) questionnaire, which originally consists of 24 questions. In order to simplify the form, we condensed the questionnaire to 9 questions concerning adverse events experienced by the children. The parents were asked to indicate whether:

1. The life or health of the child was threatened.
2. The child experienced an event in which the life or health of another person was endangered or someone died.
3. The child was assaulted physically (e.g. hitting, pushing, choking, shaking, biting, burning, forced into any type of sexual activity) or psychologically (e.g. mocking, gossiping, shouting, threatening, being rejected by someone close).
4. The child witnessed physical or psychological assault.
5. The child experienced the death of someone close.
6. The child experienced serious family problems (e.g. quarrels, conflicts, fighting, parting, alcohol problems or other addictions, emotional or mental problems of family members).
7. The child was separated from parents for an extended period.
8. The child encountered serious problems at school, such as being at the risk of failing or repeating a grade.
9. The child experienced other stressors that were not mentioned in the questionnaire.

### Genetic tests (FTO rs993960, MC4R rs177823139)

Genes and polymorphisms were selected based on their previously established relationship with vulnerability to overweight and obesity (FTO rs9939609) and changes in food intake (MC4R rs17782313). Saliva samples were collected by qualified medical personnel and DNA was extracted from the saliva following the designatedprotocol. The selected polymorphisms were genotyped using the Taq-Man single-nucleotide polymorphism (SNP) allelic discrimination method with the ABI 7900HT system (Applied Biosystems). The Real-Time PCR reaction employed commercially available TaqMan Genotyping assays for accurate results.

## Statistical methods

All statistical analyses were performed using STATISTICA 13 software. The threshold of statistical significance was set at *p* < 0.05. Analyses were conducted on tindividuals with complete information on the FTO and MC4R gene polymorphisms and body composition. For comparison analyses involving continuous dependent variables (BMI z scores, FMI z scores, FFMI z scores, and FatM z scores), we utilized the two-tailed t-test for independent groups. When comparing continuous dependent variables across more than two groups, we employed analysis of variance (ANOVA). To assess group differences in the ANOVA, Tukey's multiple comparisons test was performed. The chi-square test (χ^2^) was applied for comparative analysis of categorical variables. In order to identify the most important variables associated with BMI z scores, FMI z scores, FFMI z scores and FatM z scores the forward stepwise multiple regression was applied. Two-way ANOVA was used to assess the effects of interaction between ACEs, FTO and MC4R gene polymorphisms on the BMI z scores, FMI z scores and FatM z scores. The analysis was applied for ACE as a categorized variable (0,1,2,3 +) and for the types of ACEs (occurrence vs non-occurence). The size of effects was assessed with Cohen’s d.

## Results

### Differences according to the sex

There were no differences between the prevalence of underweight, overweight and obesity diagnosed according to IOTF between the boys and girls; however, there were differences when the diagnosis was based on body fat percentage. Obesity diagnosed according to body fat tissue was slightly more common in the boys than in the girls (8.81% vs 5.95%, χ^2^ = 11.70, *p* = 0.01) (Table 1).

**Table 1:** Prevalence of underweight, overweight and obesity diagnosed according to IOTF (Body weight status) and according to McCarthy criteria (Body fat status) for both sexes

Variable	Body weight status						Body fat status				
	n		Underweight	Proper weight	Overweight	Obesity	n	Underweight	Proper weight	Overweight	Obesity
Sex	456						454				
Boys			17	169	41	11		1	148	48	40
			3.73%	37.06%	8.99%	2.41%		0.22%	32.60%	10.57%	8.81%
Girls			28	148	29	13		0	166	24	27
			6.14%	32.46%	6.36%	2.85%		0.00%	36.56%	5.29%	5.95%
		χ^2^	5.44					**11.70**			
		p	0.14					**0.01**			

### Gene polymorphisms

There were no differences between the occurrence of underweight, overweight and obesity diagnosed according to IOTF and McCarthy norms in children with the risk allele of FTO rs9939609 or MC4R rs17782313. No significant differences were observed in the BMI z scores (F = 0.98, *p* = 0.38), FMI z scores (F = 2.26, *p* = 0.11), FFMI z scores (F = 0.19, *p* = 0.82), and FatM z scores (F = 2.63, *p* = 0.07) between the children who were homozygotes AA, heterozygotes AT and homozygotes TT of the FTO gene. However, the children who were CC homozygotes had higher FMI z scores (F = 3.11, *p* = 0.04) and FatM z scores (F = 4.09, *p* = 0.02) than the children without the risk allele (TT) of the MC4R gene. There were no differences in the BMI z scores (F = 2.72, *p* = 0.07), and FFMI z scores (F = 2.74, *p* = 0.07) (Table 2).

**Table 2:** Prevalence of underweight, overweight and obesity diagnosed according to IOTF (Body weight status) and according to McCarthy criteria (Body fat status), and association between body composition parameters and genetic factors

Variable	Body weight status						Body fat status						BMI z scores		FMI z scores		FFMI z scores		FatM z scores	
	n		Underweight	Proper weight	Overweight	Obesity	n	Underweight	Proper weight	Overweight	Obesity		$\overline{x }$	SD	$\overline{x }$	SD	$\overline{x }$	SD	$\overline{x }$	SD
FTO	456						452													
AA		All	13	67	13	4		0	69	16	12		0.20	1.22	-0.18	0.86	0.05	0.85	-0.13	0.87
			2.85%	14.69%	2.85%	0.88%		0,00%	15.2%	3.52%	2.64%									
		Girls	7	32	4	0		32	8	3	32									
			3.21%	14.68%	1.83%	0,00%		14.75%	3.69%	1.38%	14.75%									
		Boys	6	35	9	4		0	37	8	9									
			2.52%	14.71%	3.78%	1.68%		0,00%	15.61%	3.38%	3.8%									
AT		All	15	146	32	15		1	139	32	34		0.40	1.24	0.02	1.05	0.08	0.90	0.11	1.11
			3.29%	32.02%	7.02%	3.29%		0.22%	30.62%	7.05%	7.49%									
		Girls	6	69	15	10		71	11	17	71									
			2.75%	31.65%	6.88%	4.59%		32.72%	5.07%	7.83%	32.72%									
		Boys	9	77	17	5		1	68	21	17									
			3.78%	32.35%	7.14%	2.1%		0.42%	28.69%	8.86%	7.17%									
TT		All	17	104	25	5		0	106	24	21		0.29	1.20	-0.15	0.83	0.12	0.92	-0.07	0.87
			3.73%	22.81%	5.48%	1.1%		0,00%	23.35%	5.29%	4.63%									
		Girls	15	47	10	3		63	5	7	63									
			6.88%	21.56%	4.59%	1.38%		29.03%	2.3%	3.23%	29.03%									
		Boys	2	57	15	2		0	43	19	14									
			0.84%	23.95%	6.3%	0.84%		0,00%	18.14%	8.02%	5.91%									
		χ^2^	6.25					2.27				F	0.98		2.26		0.19		2.63	
		p	0.40					0.89				p	0.38		0.11		0.82		0.07	
MC4R	454						455													
CC			2	14	5	3		0	14	4	6		0.81	1.32	0.35	1.32	0.41	1.02	0.54	1.46
			0.44%	3.08%	1.1%	0.66%		0,00%	3.1%	0.88%	1.33%									
CT			13	95	23	9		0	96	22	22		0.38	1.19	-0.06	0.92	0.15	0.89	-0.02	0.92
			2.86%	20.93%	5.07%	1.98%		0,00%	21.24%	4.87%	4.87%									
TT			30	208	40	12		1	204	46	37		0.24	1.21	-0.13	0.90	0.02	0.88	-0.05	0.95
			6.61%	45.81%	8.81%	2.64%		0.22%	45.13%	10.18%	8.19%									
		χ^2^	5.30					3.63				F	2.72		**3.11**		2.74		**4.09**	
		p	0.51					073				p	0.07		**0.04**		0.07		**0.02**	
															CCvsTT^a^				CC vs CT CCvs TT^a^	

$\overline{x }$ – mean, *BMI* Body Mass Index, *FMI* Fat Mass Index, *FFMI* Fat Free Mass Index, *FatM* Fat Mass in kg

^a^ significant differences in the post hoc test

In the group of girls, obesity diagnosed on the basis of BMI was more frequent among AT heterozygotes compared to TT homozygotes (4.59% vs 1.38%, χ^2^ = 14.14, *p* = 0.03) of the FTO rs9939609 gene. BMI z scores (F = 3.17, *p* = 0.04) and FatM z scores (F = 3.41, *p* = 0.04) were higher in heterozygotes than in TT homozygotes of the FTO gene. Girls homozygous for the CC genotype had higher FatM z scores than those homozygous for the TT genotype of the MC4R gene (F = 3.24, *p* = 0.04) (Table 3).

**Table 3:** Prevalence of underweight, overweight and obesity diagnosed according to IOTF (Body weight status) and according to McCarthy criteria (Body fat status), and association between body composition parameters and genetic factors in girls

Variable			Body mass status					Body fat status						BMI z scores		FMI z scores		FFMI z scores		FatM z scores	
	n			Underweight	Proper weight	Overweight	Obesity	n	Underweight	Proper weight	Overweight	Obesity		$\overline{x }$	SD	$\overline{x }$	SD	$\overline{x }$	SD	$\overline{x }$	SD
FTO	218							217													
AA				7	32	4	0		32	8	3	32		0.09	0.96	-0.22	0.72	-0.02	0.63	-0.17	0.72
				3.21%	14.68%	1.83%	0,00%		14.75%	3.69%	1.38%	14.75%									
AT				6	69	15	10		71	11	17	71		0.44	1.17	0.11	1.06	0.08	0.75	0.18	1.11
				2.75%	31.65%	6.88%	4.59%		32.72%	5.07%	7.83%	32.72%									
TT				15	47	10	3		63	5	7	63		0.03	1.18	-0.24	0.83	0.07	0.95	-0.15	0.91
				6.88%	21.56%	4.59%	1.38%		29.03%	2.3%	3.23%	29.03%									
		χ^2^		**14.14**					7.79				F	**3.17**		**3.75**		0.28		**3.41**	
		p		**0.03**					0.10				p	**0.04**		**0.03**		0.76		**0.03**	
														AT vs TT^a^		no differences^a^				AT vs TT^a^	
MC4R	218							217													
CC				2	8	2	2		10	1	3	10		0.62	1.44	0.49	1.51	0.26	1.07	0.64	1.69
				0.92%	3.67%	0.92%	0.92%		4.61%	0.46%	1.38%	4.61%									
CT				10	50	7	5		55	9	8	55		0.26	1.16	-0.08	092	0.12	0.87	-0.05	0.92
				4.59%	22.94%	3.21%	2.29%		25.35%	4.15%	3.69%	25.35%									
TT				16	90	20	6		101	14	16	101		0.18	1.11	-0.13	0.86	0.002	0.73	-0.04	0.90
				7.34%	41.28%	9.17%	2.75%		46.54%	6.45%	7.37%	46.54%									
		χ^2^		3.63					1.42				F	0.93		2.81		1.00		**3.24**	
		p		0.73					0.84				p	0.40		0.06		0.40		**0.04**	
																				CC vs TT^a^	

$\overline{x }$ – mean, *BMI* Body Mass Index, *FMI* Fat Mass Index, *FFMI* Fat Free Mass Index, *FatM* Fat Mass in kg

^a^ significant differences in the post hoc test

In the group of boys, the incidence of underweight, overweight and obesity diagnosed on the basis of BMI and adipose tissue did not differ depending on the FTO and MC4R gene variant (Table 4). There were no differences in the BMI z scores, FMI z scores, FFMI z scores, and FatM z scores between risk homozygous, heterozygous, and homozygous children without the risk allele of the FTO and MC4R genes (Table 4).

**Table 4:** Prevalence of underweight, overweight and obesity diagnosed according to IOTF (Body weight status) and according to McCarthy criteria (Body fat status), and association between body composition parameters and genetic factors in boys

Variable	Body mass status						Body fat status						BMI z scores		FMI z scores		FFMI z scores		FatM z scores	
	n		Underweight	Proper weight	Overweight	Obesity	n	Underweight	Proper weight	Overweight	Obesity		$\overline{x }$	SD	$\overline{x }$	SD	$\overline{x }$	SD	$\overline{x }$	SD
FTO	238						217													
AA			7	32	4	0		0	37	8	9		0.29	1.40	-0.14	0.96	0.10	0.99	-0.09	0.98
			3.21%	14.68%	1.83%	0,00%		0,00%	15.61%	3.38%	3.8%									
AT			6	69	15	10		1	68	21	17		0.37	1.31	-0.06	1.03	0.07	1.02	0.04	1.10
			2.75%	31.65%	6.88%	4.59%		0.42%	28.69%	8.86%	7.17%									
TT			15	47	10	3		0	43	19	14		054	1.18	-0.07	0.82	0.16	0.91	0.003	0.82
			6.88%	21.56%	4.59%	1.38%		0,00%	18.14%	8.02%	5.91%									
		χ^2^	6.02					3.80				F	0.65		0.15		0.18		0.33	
		p	0.42					0.70				p	0.52		0.86		0.84		0.72	
MC4R	236						235													
CC			0	6	3	1		0	4	3	3		1.08	1.14	0.16	1.03	0.63	0.94	0.41	1.12
			0,00%	2.54%	1.27%	0.42%		0,00%	1.7%	1.28%	1.28%									
CT			3	45	16	4		0	41	13	14		0.51	1.22	-0.04	0.93	0.17	0.92	0.01	0.92
			1.27%	19.07%	6.78%	1.69%		0,00%	17.45%	5.53%	5.96%									
TT			14	118	20	6		1	103	32	21		0.29	1.30	-0.14	0.93	0.03	0.99	-0.05	0.99
			5.93%	50,00%	8.47%	2.54%		0.43%	43.83%	13.62%	8.94%									
		χ^2^	8.36					4.85				F	2.23		0.66		2.05		1.07	
		p	0.21					056				p	0.11		0.52		0.13		0.34	

$\overline{x }$ – mean, *BMI* Body Mass Index, *FMI* Fat Mass Index, *FFMI* Fat Free Mass Index, *FatM* Fat Mass in kg

### Socioeconomic factors

The type of family (χ^2^ = 9.08, *p* = 0.44), place of residence (χ^2^ = 7.26, *p* = 0.22), subjective assessment of economic status (χ^2^ = 4.52, *p* = 0.61) were not associated with the prevalence of underweight, overweight and obesity. The type of family was not associated with the BMI z scores (F = 0.20, *p* = 0.90), FMI z scores (F = 0.36, *p* = 0.78), FFMI z scores (F = 0.05, *p* = 0.99) or FatM z scores (F = 0.26, *p* = 0.85). There were no differences in the BMI z scores (F = 1.75, *p* = 0.18), FMI z scores (F = 1.28, *p* = 0.28), FFMI z scores (F = 0.55, *p* = 0.56) or FatM z scores (F = 1.37, *p* = 0.25) depending on the place of residence. The parental subjective assessment of the economic situation of the family was not related to the BMI z scores (F = 0.53, *p* = 0.59), FMI z scores (F = 0.31, *p* = 0.73), FFMI z scores (F = 0.90, *p* = 0.41) or FatM z scores (F = 0.37, *p* = 0.69) (Table 5).

**Table 5:** Prevalence of underweight, overweight and obesity diagnosed according to IOTF (Body weight status) and according to McCarthy criteria (Body fat status), and association between body composition parameters and socioeconomic factors

Variable	Body weight status						Body fat status						BMI z scores		FMI z scores				FFMI z scores			FatM z scores	
	n		Underweight	Proper weight	Overweight	Obesity	n	Underweight	Proper weight	Overweight	Obesity		$\overline{x }$	SD	$\overline{x }$		SD		$\overline{x }$		SD	$\overline{x }$	SD
Type of family																							
	444						442																
Both biological parents			31	260	54	16		0	252	58	49		0.30	1.17	-0.11		0.91		0.06		0.86	-0.03	0.94
			6.98%	58.56%	12.16%	3.60%		0.00%	57.01%	13.12%	11.09%												
Biological parents with partner			6	18	6	1		1	20	5	5		0.24	1.37	-0.05		0.89		0.06		0.92	-0.04	1.02
			1.35%	4.05%	1.35%	0.23%		0.23%	4.52%	1.13%	1.13%												
Single parent			7	32	7	5		0	33	8	10		0.42	1.40	0.03		1.04		0.11		0.98	0.10	1.05
			1.58%	7.21%	1.58%	1.13%		0.00%	7.47%	1.81%	2.26%												
		χ^2^	9.08					15.16				t	0.20		0.36				0.05			0.26	
		p	0.44					0.09				p	0.90		0.78				0.99			0.85	
Place of residence																							
	427						427																
Village			7	23	3	1		0	24	4	6		0.03	1.28		-0.20		0.86		-0.03	0.91	-0.15	0.90
			1.64%	5.39%	0.70%	0.23%		0.00%	5.62%	0.94%	1.41%												
Small and medium-sized town (0–100 000 residents)			5	35	11	5		1	35	7	11		0.52	1.44		0.09		1.21		0.17	0.86	0.17	1.28
			1.17%	8.20%	2.58%	1.17%		0,23%	8.20%	1.64%	2.58%												
Large city (> 100 000 residents)			31	240	50	16		0	237	57	45		0.30	1.16		-0.10		0.88		0.06	0.88	-0.03	0.92
			7.26%	56.21%	11.71%	3.75%		0,00%	55.50%	13.35%	10.54%												
		χ^2^	8.26					9.79				F	1.75			1,28				0.58		1.37	
		p	0.22					0.13				p	0.18			0,28				0.56		0.25	
Parental subjective assessment of economic situation	437						438																
Bad			1	10	4	1		0	13	1	2		0.49	1.35		-0.07		0.73		0.36	1.04	0.06	0.76
			0.23%	2.28%	0.91%	0.23%		0,00%	2.97%	0.23%	0.46%												
Average			10	46	12	6		0	48	15	11		0.41	1.33		-0.01		0.98		0.04	0.85	0.07	1.00
			2.28%	10.48%	2.73%	1.37%		0,00%	10.96%	3.42%	2.51%												
Good			34	248	51	16		1	242	54	51		0.28	1.20		-0.10		0.94		0.07	0.88	-0.03	0.98
			7.74%	56.49%	11.62%	3.64%		0.23%	55.25%	12.33%	11.64%												
		χ^2^	4.52					2.7				F	0.53			0.31				0.90		0.37	
		p	0.61					0.85				p	0.59			0.73				0.41		0.69	

$\overline{x }$ – mean, *BMI* Body Mass Index, *FMI* Fat Mass Index, *FFMI* Fat Free Mass Index, *FatM* Fat Mass in kg

### Parental body weight status

The children whose mothers had their body mass higher before pregnancy were diagnosed with overweight (IOTF) and obesity (McCarthy criteria) more often than the children whose mothers had a proper weight before pregnancy (54.55% vs 13.37%, χ^2^ = 34.32, *p* < 0.001; 36.36% vs 12.99%, χ^2^ = 20.47, *p* = 0.02, respectively). Maternal current BMI was associated with the body weight status in the children. The children of mothers who experienced obesity had higher risk of being overweight compared to the children of the mothers with a healthy weight (34.38% vs 11.45%, χ^2^ = 34.92, *p* < 0.001). The children whose mothers suffered from obesity before pregnancy had higher BMI z scores than the children of mothers who had underweight (1.35 vs -0.06) or had a proper body weight before pregnancy (1.35 vs 0.27, F = 5.11, *p* < 0.001). They also had higher FMI z scores than the children of mothers who had a proper weight before pregnancy (0.59 vs -0.14, F = 3.28, *p* = 0.02). The children of mothers who experienced obesity had higher BMI z scores (0.92 vs 0.17, F = 6.23, *p* < 0.001), FMI z scores (0.34 vs -0.18, F = 4.48, *p* = 0.004), FFMI z scores (0.43 vs -0.04, F = 6.68, *p* < 0.001) and FatM z scores (0.42 vs -0.12, F = 4.83, *p* = 0.002) than the children of the mothers with a proper body weight. The fathers with obesity were more likely to have children with obesity than the fathers with a proper body weight (13.92% vs 2.29%, χ^2^ = 27.88, *p* < 0.001). The children of fathers suffering from overweight or obesity had higher BMI z scores (F = 13.44, *p* < 0.001), FMI z scores (F = 9.48, *p* < 0.001), FFMI z scores (F = 10.36, *p* < 0.001) and FatM z scores (F = 8.50, *p* < 0.001) than the children whose fathers had a proper body weight (Table 6).

**Table 6:** Prevalence of underweight, overweight and obesity diagnosed according to IOTF (Body weight status) and according to McCarthy criteria (Body fat status), and association between body composition parameters and parental body weight status

Variable	Body weight status						Body fat status						BMI z scores		FMI z scores		FFMI z scores		FatM z scores	
	n		Underweight	Proper weight	Overweight	Obesity	n	Underweight	Proper weight	Overweight	Obesity		$\overline{x }$	SD	$\overline{x }$	SD	$\overline{x }$	SD	$\overline{x }$	SD
Maternal BMI before pregnancy			426				426													
Underweight			10	22	7	0		1	27	5	6		-0.06	1.38	-0.19	0.90	-0.19	0.91	-0.16	0.96
			2.35%	5.16%	1.64%	0,00%		0.23%	6.34%	1.17%	1.41%									
Proper weight			33	237	44	15		0	237	51	43		0.27	1.19	-0.14	0.89	0.07	0.87	-0.06	0.91
			7.75%	55.63%	10.33%	3.52%		0,00%	55.63%	11.97%	10.09%									
Overweight			1	35	6	5		0	31	8	6		0.59	1.04	0.11	1.06	0.16	0.82	0.17	1.15
			0.23%	8.22%	1.41%	1.17%		0,00%	7.28%	1.88%	1.41%									
Obesity			0	4	6	1		0	3	4	4		1.35	1.18	0.59	0.87	0.53	1.21	0.66	0.84
			0,00%	0.94%	1.41%	0.23%		0,00%	0.7%	0.94%	0.94%									
		χ^2^	**34.32**					**20.47**				F	**5.11**		**3.28**		2.34		**3.07**	
		P	** < 0.001**					**0.01**				p	** < 0.001**		**0.02**		0.07		**0.03**	
													underweight vs obesity, proper weight vs obesity^a^		proper weight vs obesity^a^				no differences^a^	
Maternal current BMI	431						432													
Underweight			4	6	4	0		0	10	2	2		0.03	1.51	-0.16	1.19	-0.03	0.90	-0.02	1.26
			0.93%	1.39%	0.93%	0,00%		0,00%	2.31%	0.46%	0.46%									
Proper weight			35	218	34	10		1	212	48	37		0.17	1.16	-0.18	0.85	-0.04	0.82	-0.12	0.86
			8.12%	50.58%	7.89%	2.32%		0.23%	49.07%	11.11%	8.56%									
Overweight			4	60	16	8		0	61	13	14		0.61	1.21	0.09	1.03	0.36	0.98	0.19	1.12
			0.93%	13.92%	3.71%	1.86%		0,00%	14.12%	3.01%	3.24%									
Obesity			1	16	11	4		0	18	7	7		0.92	1.26	0.34	1.03	0.43	0.96	0.42	1.10
			0.23%	3.71%	2.55%	0.93%		0,00%	4.17%	1.62%	1.62%									
		χ^2^	**34.92**					4.35				F	**6,23**		**4.48**		**6.68**		**4,83**	
		P	** < 0.001**					0.89				p	** < 0,001**		**0.004**		** < 0.001**		**0.002**	
													proper weight vs overweight, proper weight vs obesity^a^		proper weight vs obesity^a^		proper weight vs overweight, proper weight vs obesity^a^		proper weight vs overweight, proper weight vs obesity^a^	
Paternal current BMI	408						408													
Proper weight			16	97	15	3		104	16	12	104		0.02	1.09	-0.30	0.83	-0.09	0.79	-0.21	0.89
			3.93%	23.83%	3.69%	0.74%		25.55%	3.93%	2.95%	25.55%									
Overweight			22	137	33	5		135	30	31	135		0.25	1.16	-0.14	0.77	0.04	0.87	-0.07	0.81
			5.41%	33.66%	8.11%	1.23%		33.17%	7.37%	7.62%	33.17%									
Obesity			1	52	15	11		47	19	13	47		0.99	1.13	0.35	1.15	0.51	0.79	0.43	1.17
			0.25%	12.78%	3.69%	2.7%		11.55%	4.67%	3.19%	11.55%									
		χ^2^	**27.88**					0.34				F	**13.44**		**9.48**		**10.36**		**8.50**	
		P	** < 0.001**					0.11				p	** < 0.001**		** < 0.001**		** < 0.001**		** < 0.001**	
													proper weight vs obesity, overweight vs obesity^a^		proper weight vs obesity, overweight vs obesity^a^		proper weight vs obesity, overweight vs obesity^a^		proper weight vs obesity, overweight vs obesity^a^	

$\overline{x }$ – mean, *BMI* Body Mass Index, *FMI* Fat Mass Index, *FFMI* Fat Free Mass Index, *FatM* Fat Mass in kg

^a^ significant differences in the post hoc test

### Parental education level

The mothers with a university education were more likely to have children with a healthy body weight compared to the mothers with a vocational education (74.01% vs 56.52%, χ = 29.64, *p* < 0.001). Conversely, the mothers with a vocational education had children with a higher prevalence of excessive body fat than those with a university education (28.26% vs 9.35%, χ^2^ = 20.24, *p* = 0.02). The children of mothers with a university education had lower BMI z scores (0.20 vs 0.78, F = 3.37, *p* = 0.02), FMI z scores (-0.23 vs 0.47, F = 8.72, *p* < 0.001) and FatM z scores (-0.15 vs 0.52, F = 7.20, *p* < 0.001) than the children of mothers with a vocational education. The fathers with a vocational education had children with overweight more often than the fathers with a university education (26.74%vs11.94%, χ^2^ = 18.51, *p* = 0.03). The children of fathers with a university education had lower BMI z scores (F = 3.38, *p* = 0.02), FMI z scores (F = 5.21, *p* = 0.002) and FatM z scores (F = 4.60, *p* = 0.004) than the children whose fathers had a vocational education (Table 7).

**Table 7:** Prevalence of underweight, overweight and obesity diagnosed according to IOTF (Body weight status) and according to McCarthy criteria (Body fat status), and association between body composition parameters and parental educational level

Variable	Body weight status						Body fat status						BMI z scores		FMI z scores		FFMI z scores		FatM z scores	
	n		Underweight	Proper weight	Overweight	Obesity	n	Underweight	Proper weight	Overweight	Obesity		$\overline{x }$	SD	$\overline{x }$	SD	$\overline{x }$	SD	$\overline{x }$	SD
Maternal education level	439						439													
Primary			0	4	0	1		0	3	1	1		0.73	1.37	0.06	1.11	0.34	1.08	0.16	1.19
			0,00%	0.91%	0,00%	0.23%		0,00%	0.68%	0.23%	0.23%									
Vocational			3	26	9	8		0	25	8	13		0.78	1.49	0.47	1.36	0.33	0.94	0.52	1.41
			0.68%	5.92%	2.05%	1.82%		0,00%	5.69%	1.82%	2.96%									
Secondary			15	70	18	8		1	73	14	22		0.37	1.39	0.02	1.03	0.09	1.02	0.09	1.09
			3.42%	15.95%	4.1%	1.82%		0.23%	16.63%	3.19%	5.01%									
University			26	205	41	5		0	204	48	26		0.20	1.06	-0.23	0.73	0.03	0.81	-0.15	0.76
			5.92%	46.7%	9.34%	1.14%		0,00%	46.47%	10.93%	5.92%									
		χ^2^	**29.64**					**20.24**				F	**3.37**		**8.72**		1.74		**7.20**	
		P	**0.001**					**0.02**				p	**0.02**		** < 0.001**		0.16		** < 0.001**	
													secondary vs higher^a^		secondary vs vocational, secondary vs higher^a^				Secondary vs vocational, secondary vs higher^a^	
Paternal education level	409						422													
Primary			11	7	2	1		0	7	1	3		0.53	1.39	0.18	1.19	0.18	1.26	0.20	1.18
			0.24%	1.65%	0.47%	0.24%		0,00%	1.66%	0.24%	0.71%									
Vocational			8	49	23	6		0	51	15	19		0.60	1.32	0.15	1.00	0.22	0.88	0.25	1.08
			1.89%	11.58%	5.44%	1.42%		0,00%	12.09%	3.55%	4.5%									
Secondary			11	88	17	9		0	88	18	19		0.40	1.24	-0.01	1.04	0.16	0.91	0.05	1.05
			2.6%	20.8%	4.02%	2.13%		0,00%	20.85%	4.27%	4.5%									
University			19	154	24	4		0	148	35	18		0.15	1.04	-0.26	0.71	-0.02	0.81	-0.17	0.74
			4.49%	36.41%	5.67%	0.95%		0,00%	35.07%	8.29%	4.27%									
		χ^2^	**18.51**					11.95				F	**3.38**		**5.21**		2.11		**4.60**	
		P	**0.03**					0.22				p	**0.02**		**0.002**		0.10		**0.004**	
													secondary vs higher^a^		secondary vs higher^a^				secondary vs higher^a^	

$\overline{x }$ – mean, *BMI* Body Mass Index, *FMI* Fat Mass Index, *FFMI* Fat Free Mass Index, *FatM* Fat Mass in kg

^a^ significant differences in the post hoc test

### Lifestyle

Eating at least 3 meals and 1 snack per day was not associated with the prevalence of abnormal body weight, body fat status or body composition parameters (*p* > 0.05). The children who exercised at least 3 h 3 days per week had a proper body weight more frequently than the children who exercised less often (76.84% vs 67,41%, χ^2^ = 8.08, *p* = 0.04). Spending time in front of the screen was not associated with the prevalence of an abnormal body weight or body fat status (χ^2^ = 7.38, *p* = 0.06; χ^2^ = 2.86, *p* = 0.41, respectively). The children who spent more than 2 h in front of the screen had higher BMI z scores (0.42 vs 0.18, t = 2.03, *p* = 0.04), FMI z scores (0.01 vs -0.20, t = 2.44, *p* = 0.02), and FatM z scores (0.11 vs -0.16, t = 2.92, *p* = 0.003). Sleep length was not associated with underweight, overweight and obesity diagnosed according to IOTF and McCarthy norms (χ^2^ = 0.37, *p* = 0.94; χ^2^ = 0.41, *p* = 0.93, respectively) or body composition parameters (*p* > 0.05) (Table 8).

**Table 8:** Prevalence of underweight, overweight and obesity diagnosed according to IOTF (Body weight status) and according to McCarthy criteria (Body fat status), and association between body composition parameters and lifestyle factors

Variable	Body weight status						Body fat status						BMI z scores		FMI z scores		FFMI z scores		FatM z scores	
	n		Underweight	Proper weight	Overweight	Obesity	n	Underweight	Proper weight	Overweight	Obesity		$\overline{x }$	SD	$\overline{x }$	SD	$\overline{x }$	SD	$\overline{x }$	SD
Eating behaviour	454						454													
Bad			24	167	44	16		1	165	44	40		0.39	1.25	-0.05	0.89	0.14	0.89	0.02	0.93
			5.29%	36.78%	9.69%	3.52%		0.22%	36.34%	9.69%	8.81%									
Good			21	148	26	8		0	149	28	27		0.23	1.18	-0.12	1.00	0.02	0.89	-0.03	1.05
			4.63%	32.6%	5.73%	1.76%		0,00%	32.82%	6.17%	5.95%									
		χ^2^	3.61					3.27				t	1.40		0.79		1.40		0.59	
		P	0.31					0.35				p	0.16		0.43		0.16		0.55	
Physical activity	454																			
Bad			42	242	58	17		1	244	61	53		0.31	1.23	-0.08	0.93	0.05	0.88	-0.02	0.96
			9.25%	53.3%	12.78%	3.74%		0.22%	53.74%	13.44%	11.67%									
Good			3	73	12	7		0	70	11	14		0.37	1.21	-0.08	0.98	0.22	0.94	0.06	1.08
			0.66%	16.08%	2.64%	1.54%		0,00%	15.42%	2.42%	3.08%									
		χ^2^	**8.08**					2.01				t	-0.46		0.08		-1.63		-0.66	
		P	**0.04**					0.57				p	0.64		0.94		0.10		0.51	
Screen time	454						454													
> 2 h			23	180	51	13		0	184	39	43		0.42	1.25	0.01	1.02	0.14	0.90	0.11	1.09
			5.07%	39.65%	11.23%	2.86%		0,00%	40.53%	8.59%	9.47%									
≤ 2 h			22	135	19	11		1	130	33	24		0.18	1.17	-0.20	0.81	0.01	0.88	-0.16	0.79
			4.85%	29.74%	4.19%	2.42%		0.22%	28.63%	7.27%	5.29%									
		χ^2^	7.38					2.86				t	2.03		2.44		1.57		2.92	
		P	0.06					0.41				p	0.04		0.02		0.12		0.003	
Sleep length	432						432													
< 9 h			35	267	59	19		1	265	61	53		0.34	1.21	-0.08	0.93	0.10	0.87	-0.01	0.97
			8.1%	61.81%	13.66%	4.4%		0.23%	61.34%	14.12%	12.27%									
≥ 9 h			6	35	8	3		0	38	7	7		0.24	1.24	-0.13	0.89	0.01	0.99	0.01	0.99
			1.39%	8.1%	1.85%	0.69%		0,00%	8.8%	1.62%	1.62%									
		χ^2^	0.37					0.41				t	0.52		0.37		0.68		-0.14	
		P	0.94					0.93				p	0.60		0.71		0.50		0.89	

$\overline{x }$ – mean, *BMI* Body Mass Index, *FMI* Fat Mass Index, *FFMI* Fat Free Mass Index, *FatM* Fat Mass in kg, *ACE* Adverse Childhood Events

### Perinatal factors

The children of mothers who had at least one disease (hypertension, diabetes, thyroid diseases, kidney diseases or heart failure) before pregnancy had obesity diagnosed according to body fat tissue less often (12.5% vs 21.19%, χ = 8.66, *p* = 0.03) than the children of mothers without the diseases. The children with a birthweight above 4000 g had a higher risk of being overweight in childhood than the children with a birthweight 2500-4000 g (23.33% vs 14.40%, χ^2^ = 16.25, *p* = 0.02). Birthweight had no association with the body fat status. The pregnancy duration was not associated with the BMI z scores (t = 0.11, *p* = 0.90), FMI z scores (t = 0.24, *p* = 0.79), FFMI z scores (t = 0.10, *p* = 0.90) or FatM z scores (t = 0.42, *p* = 0.66). Prenatal stress had no impact on the BMI z scores (t = -1.31, *p* = 0.19), FMI z scores (t = -0.40, *p* = 0.69), FFMI z scores (t = -0.89, *p* = 0.38) or FatM z scores (t = -0.30, *p* = 0.76). The children who had a twin had the BMI z scores (0.33 vs -0.31, t = 2.31, *p* = 0.02) and the FatM z scores (-0.01 vs -0.43, t = 1.97, *p* = 0.04) lower than the single born children. The type of delivery had an impact on the FMI z scores and FatM z scores. The children born vaginally had the FMI z scores (-0.02 vs -0.27, t- = 2.64, *p* = 0.01) lower than the children delivered by a c-section, but they had higher FatM z scores (0.04 vs -0.16, t = 2.06, *p* = 0.04). Breastfeeding was not related to the BMI z scores (t = -0.40, *p* = 0.69), FMI z scores (t = -0.35, *p* = 0.73), FFMI z scores (t = -0.80, *p* = 0.43), or FatM z scores (t = -0.24, *p* = 0.81). The children of the mothers with exceeded weight gain during pregnancy were more likely to suffer from obesity (12.2 vs 4.5, χ^2^ = 12.79, *p* = 0.01). Moreover, they had higher BMI z scores (0.82 vs 0.27, t = 2.77, *p* = 0.01), FMI z scores (0.33 vs -0.12, t = 2.96, *p* < 0.01), FFMI z scores (0.38 vs 0.05, t = 2.29, *p* = 0.02) and FatM z scores (0.53 vs -0.06, t = 3.76, *p* < 0.01) (Table 9).

**Table 9:** Prevalence of underweight, overweight and obesity diagnosed according to IOTF (Body weight status) and according to McCarthy criteria (Body fat status), and association between body composition parameters and perinatal factors

Variable	Body weight status						Body fat status						BMI z scores		FMI z scores		FFMI z scores		FatM z scores	
	n		Underweight	Proper weight	Overweight	Obesity	n	Underweight	Proper weight	Overweight	Obesity		$\overline{x }$	SD	$\overline{x }$	SD	$\overline{x }$	SD	$\overline{x }$	SD
Pregnancy duration	425						423													
< 37 week			1	19	6	4		0	40	9	9		0.35	1.14	-0.13	0.90	0.13	0.77	-0.08	0.87
			0.24%	4.47%	1.41%	0.94%		0,00%	9.46%	2.13%	2.13%									
37–42 week			40	276	59	19		1	250	56	56		0.34	1.23	-0.05	0.95	0.08	0.91	0.03	1.00
			9.41%	64.94%	13.88%	4.47%		0.24%	59.1%	13.24%	13.24%									
> 42 week			0	1	0	0		0	0	2	0		0.74	0.59	0.20	0.20	0.01	0.76	0.36	0.03
			0,00%	0.24%	0,00%	0,00%		0,00%	0,00%	0.47%	0,00%									
		χ^2^	6.15					10.84				F	0.11		0.24		0.10		0.42	
		p	0.41					0.09				p	0.90		0.79		0.90		0.66	
At least one disease during pregnancy	454						454													
No			27	223	46	13		0	217	53	38		0.31	1.14	-0.12	0.82	0.08	0.88	-0.06	0.85
			5.95%	49.12%	10.13%	2.86%		0,00%	47.8%	11.67%	8.37%									
Yes			18	92	24	11		1	97	19	29		0.36	1.39	0.02	1.15	0.10	0.92	0.13	1.22
			3.96%	20.26%	5.29%	2.42%		0.22%	21.37%	4.19%	6.39%									
		χ^2^	4,74					7.24				t	-0.40		-1.54		-0.24		-1.93	
		p	0.19					0.06				p	0.69		0.12		0.81		0.05	
At least one disease before pregnancy	456						533													
No			33	241	48	16		0	237	57	42		0.30	1.19	-0.12	0.89	0.06	0.87	-0.05	0.92
			7.24%	52.85%	10.53%	3.51%		0,00%	52.2%	12.56%	9.25%									
Yes			12	76	22	8		1	77	15	25		0.40	1.31	0.04	1.06	0.14	0.97	0.16	1.14
			2.63%	16.67%	4.82%	1.75%		0.22%	16.96%	3.3%	5.51%									
		χ^2^	2.43					**8.66**				t	-0.76		-1.52		-0.75		-2.00	
		p	0.49					**0.03**				p	0.45		0.13		0.45		0.05	
Prenatal stress	454						440													
No			35	229	47	17		1	237	44	47		0.28	1.22	-0.09	0.95	0.06	0.90	-0.01	1.00
			7.71%	50.44%	10.35%	3.74%		0.22%	52.2%	9.69%	10.35%									
Yes			10	86	23	7		0	77	28	20		0.44	1.23	-0.05	0.93	0.14	0.87	0.02	0.95
			2.2%	18.94%	5.07%	1.54%		0,00%	16.96%	6.17%	4.41%									
		Χ	1.65					6.64				t	-1.31		-0.40		-0.89		-0.30	
		p	0.64					0.08				p	0.19		0.69		0.38		0.76	
Type of pregnancy	437						435													
Single			39	294	65	19		1	288	68	58		0.33	1.20	-0.08	0.92	0.08	0.88	-0.01	0.95
			8.92%	67.28%	14.87%	4.35%		0.23%	66.21%	15.63%	13.33%									
Twin			5	12	1	2		0	17	1	2		-0.31	1.28	-0.46	0.82	-0.10	0.77	-0.43	0.72
			1.14%	2.75%	0.23%	0.46%		0,00%	3.91%	0.23%	0.46%									
		χ^2^	7.53					2.49				t	**2.31**		1.82		0.89		**1.97**	
		p	0.06					0.48				p	**0.02**		0.07		0.37		**0.04**	
Type of delivery	434						434													
Vaginal			32	207	51	18		1	204	55	50		0.35	1.26	-0.02	0.99	0.10	0.93	0.04	1.02
			7.34%	47.48%	11.7%	4.13%		0.23%	46.79%	12.61%	11.47%									
C-section			11	98	16	3		0	99	16	11		0.21	1.07	-0.27	0.67	0.02	0.75	-0.16	0.73
			2.52%	22.48%	3.67%	0.69%		0,00%	22.71%	3.67%	2.52%									
		χ^2^	4.70					7.41				t	1.11		**2.64**		0.83		**2.06**	
		p	0.20					0.06				p	0.27		**0.01**		0.41		**0.04**	
Birthweight	441						441													
< 2500			6	10	2	2		0	16	2	2		-0.20	1.49	-0.32	0.95	0.01	1.05	-0.29	0.83
			1.36%	2.27%	0.45%	0.45%		0,00%	3.63%	0.45%	0.45%									
2500–4000			36	255	52	18		1	250	60	51		0.30	1.21	-0.08	0.92	0.06	0.89	-0.01	0.97
			8.16%	57.82%	11.79%	4.08%		0.23%	56.69%	13.61%	11.56%									
> 4000			2	42	14	2		0	40	10	9		0.54	1.12	-0.09	0.92	0.24	0.82	0.07	0.95
			0.45%	9.52%	3.17%	0.45%		0,00%	9.07%	2.27%	2.04%									
		χ^2^	**16.25**					3.38				t	2.85		0.64		1.18		1.06	
		p	**0.02**					0.97				p	0.06		0.53		0.31		0.35	
Breastfeeding	443						441													
Yes			40	283	60	20		1	282	64	54		0.30	1.22	-0.10	0.92	0.07	0.89	-0.02	0.96
			9.03%	63.88%	13.54%	4.51%		0.23%	63.95%	14.51%	12.24%									
No			4	26	8	2		0	25	8	7		0.38	1.20	-0.05	0.83	0.18	0.83	0.02	0.87
			0.9%	5.87%	1.81%	0.45%		0,00%	5.67%	1.81%	1.59%									
		χ^2^	0.76					1.21				t	-0.40		-0.35		-0.80		-0.24	
		p	0.87					0.75				p	0.69		0.73		0.43		0.81	
Siblings	434						434													
Yes			33	238	47	16		1	238	51	43		0.27	1.18	-0.13	0.91	0.05	0.85	-0.05	0.93
			7.6%	54.84%	10.83%	3.69%		0.23%	54.84%	11.75%	9.91%									
No			11	66	19	4		0	68	17	16		0.35	1.25	-0.03	0.93	0.10	0.90	0.04	1.01
			2.53%	15.21%	4.38%	0.92%		0,00%	15.67%	3.92%	3.69%									
		χ^2^	1.73					1.09				t	-0.60		-0.89		-0.57		-0.73	
		p	0.63					0.78				p	0.55		0.37		0.57		0.46	
Weight gain during pregnancy	434						434													
Exceeded			2	22	12	5		0	22	9	9		0.82	1.30	0.33	1.12	0.38	1.17	0.53	1.22
			0.46%	5.07%	2.76%	1.15%		0,00%	5.07%	2.07%	2.07%									
Non-exceeded			42	279	54	18		1	277	59	57		0.27	1.21	-0.12	0.91	0.05	0.85	-0.06	0.93
			9.68%	64.29%	12.44%	4.15%		0.23%	63.82%	13.59%	13.13%									
		χ^2^	**12.79**					4.19				t	**2.77**		**2.96**		**2.29**		**3.76**	
		p	**0.01**					0.24				p	**0.01**		** < 0.01**		**0.02**		** < 0.001**	

$\overline{x }$ – mean, *BMI* Body Mass Index, *FMI* Fat Mass Index, *FFMI* Fat Free Mass Index, *FatM* Fat Mass in kg, *ACE* Adverse Childhood Events

### Adverse childhood experiences

The number of experienced events was associated with overweight diagnosed according to IOTF (χ^2^ = 17.27, *p* = 0.04). However, the experience of stressors was not associated with the body fat status (χ^2^ = 13.0, *p* = 0.16). The children who had been exposed to family conflicts had obesity more often than the children who had not experienced that stressor (9.46% vs 3.70%, χ^2^ = 12.44, *p* = 0.01). The children who had witnessed violence had excessive body fat more often than the children who had not experienced the stressor (18.18% vs 13.55%, χ^2^ = 12.63, *p* = 0.01). The children who had experienced death of someone close were diagnosed overweight according to body fat tissue more often than the children who had not experienced the stressor (22.67% vs 13.96%, χ^2^ = 8.50, *p* = 0.03). The children who had been separated from their parents were more often diagnosed underweight according to BMI (8.17% vs 19.30%, χ^2^ = 8.44, *p* = 0.04) and more often obese according to body fat tissue (12.81 vs 19.30%, χ^2^ = 8.35, *p* = 0.04) compared to the children who had not been separated from their parents. Various types of stressors were not associated with the body composition parameters (Table 10).

**Table 10:** Prevalence of underweight, overweight and obesity diagnosed according to IOTF (Body weight status) and according to McCarthy criteria (Body fat status), and association between body composition parameters and ACE

Variable	Body weight status						Body fat status						BMI z scores		FMI z scores		FFMI z scores		FatM z scores	
	n		Underweight	Proper weight	Overweight	Obesity	n	Underweight	Proper weight	Overweight	Obesity		$\overline{x }$	SD	$\overline{x }$	SD	$\overline{x }$	SD	$\overline{x }$	SD
ACE	454						454													
0			18	189	35	8		0	180	36	35		0.26	1.12	-0.14	0.88	0.04	0.84	-0.07	0.92
			3.96%	41.63%	7.71%	1.76%		0,00%	39.65%	7.93%	7.71%									
1			13	67	19	10		0	74	20	15		0.49	1.29	0.03	0.99	0.18	0.91	0.14	1.03
			2.86%	14.76%	4.19%	2.2%		0,00%	16.3%	4.41%	3.3%									
2			8	28	8	5		0	29	8	11		0.42	1.52	0.04	1.21	0.18	1.06	0.16	1.30
			1.76%	6.17%	1.76%	1.1%		0,00%	6.39%	1.76%	2.42%									
3 +			6	31	8	1		1	31	8	6		0.14	1.27	-0.10	0.80	0.00	0.93	-0.10	0.83
			1.32%	6.83%	1.76%	0.22%		0.22%	6.83%	1.76%	1.32%									
		χ^2^	**17,27**					13.00				F	1.33		1.14		0.97		1.80	
		p	**0.04**					0.16				p	0.27		0.33		0.41		0.15	
Life/health threat	426						426													
No			34	274	63	18		1	273	60	55		0.33	1.19	-0.08	0.92	0.07	0.86	0.00	0.96
			7.98%	64.32%	14.79%	4.23%		0.23%	64.08%	14.08%	12.91%									
Yes			7	24	4	2		0	27	6	4		0.09	1.35	-0.24	0.90	0.04	1.05	-0.15	0.95
			1.64%	5.63%	0.94%	0.47%		0,00%	6.34%	1.41%	0.94%									
		χ^2^	4.45					0.42				t	1.15		1.00		0.21		0.92	
		p	0.22					0.95				p	0.25		0.32		0.83		0.36	
Life/health threat witness	426																			
No			41	270	62	19		1	279	60	53		0.29	1.22	-0.10	0.93	0.07	0.90	-0.02	0.97
			9.62%	63.38%	14.55%	4.46%		0.23%	65.49%	14.08%	12.44%									
Yes			0	28	5	1		0	21	6	6		0.53	0.89	-0.04	0.72	0.11	0.62	0.03	0.78
			0,00%	6.57%	1.17%	0.23%		0,00%	4.93%	1.41%	1.41%									
		χ^2^	4.63					0.97				t	-1.12		-0.37		-0.23		-0.29	
		p	0.20					0.81				p	0.27		0.71		0.82		0.77	
Violence victim	426						426													
No			35	274	63	17		1	272	60	56		0.33	1.17	-0.09	0.91	0.07	0.85	-0.01	0.96
			8.22%	64.32%	14.79%	3.99%		0.23%	63.85%	14.08%	13.15%									
Yes			6	24	4	3		0	28	6	3		0.04	1.45	-0.18	0.97	0.07	1.16	-0.12	0.99
			1.41%	5.63%	0.94%	0.7%		0,00%	6.57%	1.41%	0.7%									
		χ^2^	3.61					1.23				t	1.41		0.57		0.04		0.72	
		p	0.31					0.75				p	0.16		0.57		0.97		0.47	
Violence witness	424						424													
No			37	276	60	18		0	277	61	53		0.31	1.18	-0.11	0.90	0.07	0.88	-0.03	0.94
			8.73%	65.09%	14.15%	4.25%		0,00%	65.33%	14.39%	12.5%									
Yes			4	20	7	2		1	22	4	6		0.26	1.43	0.07	1.09	0.10	0.97	0.13	1.20
			0.94%	4.72%	1.65%	0.47%		0.24%	5.19%	0.94%	1.42%									
		χ^2^	1.46					**12.63**				t	0.22		-1.08		-0.16		-0.92	
		P	0.69					**0.01**				p	083		0.28		0.87		0.36	
Disease/death of someone close	426						426													
No			35	247	52	16		0	253	49	49		0.29	1.18	-0.11	0.91	0.08	0.90	-0.03	0.95
			8.22%	57.98%	12.21%	3.76%		0,00%	59.39%	11.5%	11.5%									
Yes			6	51	15	4		1	47	17	10		0.39	1.29	-0.01	0.94	0.03	0.78	0.05	0.99
			1.41%	11.97%	3.52%	0.94%		0.23%	11.03%	3.99%	2.35%									
		χ^2^	1.40					**8.50**				t	-0.65		-0.90		0.51		-0.62	
		P	0.70					**0.03**				p	0.52		0.37		0.61		0.54	
Family conflicts	425						425													
No			28	256	54	13		0	252	53	46		0.30	1.15	-0.12	0.89	0.05	0.85	-0.04	0.93
			6.59%	60.24%	12.71%	3.06%		0,00%	59.29%	12.47%	10.82%									
Yes			13	42	12	7		1	48	13	12		0.31	1.41	0.01	1.02	0.15	1.00	0.06	1.08
			3.06%	9.88%	2.82%	1.65%		0.24%	11.29%	3.06%	2.82%									
		χ^2^	**12.44**					**5.83**				t	-0.09		-1.07		-0.82		-0.79	
		P	**0.01**					**0.12**				p	0.93		0.29		0.41		0.43	
Separation from parents																				
No			30	264	57	16		0	263	57	47		0.32	1.17	-0.10	0.88	0.09	0.87	-0.02	0.92
			7.08%	62.26%	13.44%	3.77%		0,00%	62.03%	13.44%	11.08%									
Yes			11	33	9	4		1	37	8	11									
			2.59%	7.78%	2.12%	0.94%		0.24%	8.73%	1.89%	2.59%		0.20	1.42	-0.03	1.14	-0.03	0.94	0.03	1.18
		χ^2^	**8.44**					**8.35**				t	0.69		-0.55		0.99		-0.40	
		P	**0.04**					**0.04**				p	0.49		0.58		0.32		0.69	
School problems	423						423													
No			39	290	63	19		0	293	63	55		0.30	1.19	-0.10	0.92	0.06	0.87	-0.02	0.96
			9.22%	68.56%	14.89%	4.49%		0,00%	69.27%	14.89%	13,00%									
Yes			2	6	3	1		1	6	2	3		0.38	1.67	0.00	0.95	0.38	1.17	0.06	1.07
			0.47%	1.42%	0.71%	0.24%		0.24%	1.42%	0.47%	0.71%									
		χ^2^	2.36					**36.16**				t	-0.22		-0.37		-1.23		-0.28	
		p	0.50					** < 0.001**				p	0.83		0.71		0.22		0.78	
Other unspecified stressors	421						421													
No			38	284	61	19		1	286	62	53		0.30	1.19	-0.10	0.92	0.06	0.86	-0.02	0.96
			9.03%	67.46%	14.49%	4.51%		0.24%	67.93%	14.73%	12.59%									
Yes			3	10	5	1		0	12	3	4		0.41	1.60	0.03	0.95	0.26	1.26	0.14	0.97
			0.71%	2.38%	1.19%	0.24%		0,00%	2.85%	0.71%	0.95%									
		χ^2^	3.04					1.04				t	-0.38		-0.60		-0.95		-0.72	
		P	0.39					0.79				p	0.71		0.55		0.34		0.47	

$\overline{x }$-mean, *BMI* Body Mass Index, *FMI* Fat Mass Index, *FFMI* Fat Fress Mass Index, *FatM* Fat Mass in kg, *ACE* Adverse Childhood Events

### Multiple regression models for body composition parameters

Multiple regression models were prepared for each dependent variable (BMI z scores, FMI z scores, FFMI z scores, and FatM z scores) including the following independent variables: the type of family, highest body weight during pregnancy, pregnancy duration, prenatal stress, mother’s age at the child’s birth, maternal BMI before pregnancy, maternal current BMI, maternal education level, paternal current BMI, paternal education level, ACE experience, eating behaviour, physical activity, screen time, sleep length, and polymorphisms of the FTO and MC4R genes. All thes variables were included in the stepwise multiple regression model. The variables were added until the highest value of R^2^ was reached. Multiple regression was performed for the whole group and separately for boys and girls.

The variables that remained in the model for all groups, explaining 17.2% (F = 7.02, *p* < 0.001) of the variability in BMI z scores were paternal BMI, highest body weight during pregnancy, maternal age at the child’s birth, paternal education level, prenatal stress, maternal BMI before pregnancy, eating behaviour and FTO and MC4R gene polymorphisms. Among these variables, paternal BMI, highest body weight in pregnancy and maternal age at the child’s birth made the greatest contribution to the prediction of the dependent variable (ΔR^2^ = 0.085, *p* < 0.001; ΔR^2^ = 0.031, *p* = 0.16, ΔR^2^ = 0.021, *p* = 0.01, respectively). Specifically, paternal BMI was positively associated with BMI z scores (β = 0.26, *p* < 0.001), while the mother’s age at the child’s birth was negatively associated with BMI z scores (β = -0.13, *p* = 0.01) (Table 11).

**Table 11:** Regression analysis predicting BMI z scores, FMI z scores and FatM z scores in children

Variable	R2	β	F	*P*-value
Model 1: BMI z scores	0.172		7.02	< 0.001
Paternal BMI		0.26		< 0.001
Highest body weight in pregnancy		0.11		0.16
Mother's age at the child’s birth		-0.13		0.01
Father's education (Reference: higher education)		0.08		0.11
Prenatal stress at least one (Reference: 0)		0.07		0.17
Maternal BMI before pregnancy		0.11		0.15
FTO (Reference: TT)		0.07		0.18
Eating behaviour (Reference: good)		0.07		0.2
MC4R (Reference: TT)		0.05		0.31
Model 2: FMI z scores	0.138		10.24	< 0.001
Paternal BMI		0.21		< 0.001
Maternal education level (Reference: higher education)		0.18		< 0.001
Highest body weight in pregnancy		0.16		< 0.001
Mother's age at the child’s birth		-0.10		0.06
FTO (Reference: TT)		0.10		0.04
ACE at least one (Reference: 0)		0.07		0.19
Model 3: FFMI z scores	0.125		8.09	< 0.001
Paternal BMI		0.25		< 0.001
Maternal pre-pregnancy BMI		0.14		0.006
Mother's age at the child’s birth		-0.13		0.01
Eating behaviour (Reference: good)		0.08		0.15
MC4R (Reference: TT)		0.06		0.21
Paternal education level (Reference: higher education)		0.05		0.30
Model 4: FatM z scores	0.178		9.17	< 0.001
Maternal education level (Reference: higher education)		0.16		0.01
Highest body weight in pregnancy		0.21		< 0.001
Paternal BMI		0.17		< 0.001
Mother's age at the child’s birth		-0.11		0.04
FTO (Reference: TT)		0.11		0.03
Paternal education level (Reference: higher education)		0.06		0.28
ACE at least one (Reference: 0)		0.06		0.25
Eating behaviour (Reference: good)		0.06		0.27

*BMI* Body Mass Index, *FMI* Fat Mass Index, *FFMI* Fat Free Mass Index, *FatM* Fat Mass in kg

Thirteen point eight percent (13.8%) of the variance in FMI z scores was explained by paternal BMI, maternal education, highest body weight during pregnancy, maternal age at the child’s birth, FTO polymorphism, and experience of ACE. Among the variables, paternal BMI (β = 0.21, *p* < 0.001), maternal education (β = 0.18, *p* < 0.001), highest body weight in pregnancy (β = 0.16, *p* < 0.001) and FTO polymorphism (β = 0.10, *p* = 0.04) were positively connected with FMI z scores. Paternal BMI, maternal education and highest body weight during pregnancy had the greatest contribution to predicting the variable (ΔR^2^ = 0.056, *p* < 0.001; ΔR^2^ = 0.043, *p* < 0.001, ΔR^2^ = 0.025, *p* =  < 0.001, respectively) (Table 11).

The FFMI z scores were predicted by 6 variables: paternal BMI, maternal pre-pregnancy BMI, maternal age at the child’s birth, eating behaviour, MC4R gene polymorphism, and paternal education. The model explained 12.5% of the variance in the dependent variable (*p* < 0.001). Among the variables, paternal BMI, maternal pre-pregnancy BMI and maternal age at the child’s birth had the greatest contributions (ΔR^2^ = 0.075, *p* < 0.001; ΔR^2^ = 0.019, *p* = 0.006, ΔR^2^ = 0.018, *p* = 0.01, respectively). Paternal BMI (β = 0.25, *p* < 0.001) and maternal pre-pregnancy BMI (β = 0.14, *p* = 0.006) were positively associated with the dependent variable, while the maternal age at the child’s birth (β = -0.13, *p* = 0.01) showed a negative association with FFMI z scores (Table 11).

The forward regression model for the FatM z scores included maternal education, highest body weight in pregnancy, paternal BMI, maternal age at the child’s birth, FTO gene polymorphism, paternal education, experiences of ACE and eating behaviour, which explained 17.8% of the variance in FatM z scores. The FatM z scores were positively associated with maternal education (β = 0.16, *p* = 0.01), highest body weight in pregnancy (β = 0.21, *p* < 0.001), paternal BMI (β = 0.17, *p* < 0.001), and FTO polymorphism (β = 0.11, *p* = 0.03), while they were negatively associated with maternal age at the child’s birth (β = -0.11, *p* = 0.04). Maternal education, highest body weight in pregnancy and paternal BMI made the greatest contributions predicting the variable (ΔR^2^ = 0.059, *p* = 0.01, ΔR^2^ = 0.054, *p* < 0.001, ΔR^2^ = 0.028, *p* < 0.01, respectively) (Table 11).

In the group of girls, the variables that remained in the multiple regression model explaining 26% (F = 11.53, *p* < 0.001) of BMI variability in z scores were paternal BMI, highest body weight during pregnancy, FTO gene polymorphism, maternal education, and duration of pregnancy. Paternal BMI, highest body weight during pregnancy and FTO gene polymorphism had the greatest contribution to the prediction of the dependent variable (respectively: ΔR2 = 0.144, *p* < 0.001; ΔR2 = 0.07, *p* < 0.001, ΔR2 = 0.022, *p* = 0.04). These variables were positively correlated with BMI z scores (Table 12).

**Table 12:** Multiple forward regression results predicting BMI z scores, FMI z scores, FFMI z scores, and FatM z scores in girls

Variable	R2	β	F	*P*-value
Model 1: BMI z scores	0.260		11.53	< 0.001
Paternal BMI		0.329		< 0.001
Highest body weight in pregnancy		0.284		< 0.001
FTO (ref. TT)		0.142		0.04
Maternal education level (Reference: higher education)		0.131		0.05
Pregnancy duration		-0.07		0.27
Model 2: FMI z scores	0.290		7.46	< 0.001
Paternal BMI		0.25		< 0.001
Highest body weight in pregnancy		0.34		< 0.001
Maternal education level (Reference: higher education)		0.26		< 0.001
FTO (ref. TT)		0.15		0.03
Pregnancy duration		-0.11		0.10
MC4R (ref. TT)		0.089		0.19
Maternal current BMI		-0.09		0.38
Prenatal stress (ref. 0)		-0.10		0.18
ACE at least one (ref. 0)		0.09		0.21
Model 3: FFMI z scores	0.147		4.68	< 0.001
Paternal BMI		0.27		< 0.001
Highest body weight in pregnancy		0.16		0.04
Mother's age at the child’s birth		-0.10		0.18
Pregnancy duration		-0.07		0.31
Prenatal stress (ref. 0)		-0.09		0.24
Sleep duration		0.08		0.30
Model 4: FatM z scores	0.289		8.17	< 0.001
Highest body weight in pregnancy		0.40		< 0.001
Paternal BMI		0.23		0.001
Maternal education level (Reference: higher education)		0.25		< 0.001
FTO (ref. TT)		0.14		0.04
Maternal current BMI		-0.13		0.19
Pregnancy duration		-0.09		0.18
Prenatal stress (ref. 0)		-0.12		0.10
ACE at least one (ref. 0)		0.11		0.13

Twenty-nine percent (29%) of the variance in FMI scores in the girls’ group was explained by paternal BMI, highest gestational weight, maternal education, FTO polymorphism, duration of pregnancy, MC4R polymorphism, current maternal BMI, prenatal stress experience and ACE. Father's BMI (β = 0.25, *p* < 0.001), highest gestational weight (β = 0.34, *p* < 0.001), maternal education (β = 0.26, *p* < 0.001) and FTO gene polymorphism (β = 0.15, *p* = 0.03) were positively associated with FMI z scores and had the greatest impact on the prediction of the dependent variable (ΔR2 = 0.102, *p* < 0.001; ΔR2 = 0.067, *p* < 0.001, ΔR2 = 0.066, *p* =  < 0.001, ΔR2 = 0.002 *p* = 0.03 respectively) (Table 12).

The girls' FFMI scores were predicted based on six variables: father's BMI, highest gestational weight, mother's age at birth, duration of pregnancy, prenatal stress experience and sleep duration. The model explained 14.7% of the variance of the dependent variable (*p* < 0.001). Paternal BMI and highest body weight during pregnancy showed the strongest influence (ΔR2 = 0.096, *p* < 0.001; ΔR2 = 0.024, *p* = 0.04, respectively). Paternal BMI (β = 0.27, *p* < 0.001) and highest gestational weight (β = 0.16, *p* = 0.04) were positively associated with the dependent variable (Table 12).

The stepwise regression model for FatM z scores in the girls’ group included highest gestational weight, paternal BMI, maternal education, FTO gene polymorphism, current maternal BMI, duration of pregnancy, experience of prenatal stress and ACE, which explained 28.9% of the variance in FatM z scores. FatM scores were positively associated with highest gestational weight (β = 0.40, *p* < 0.001), paternal BMI (β = 0.23, *p* = 0.001), maternal education (β = 0.25, < 0.001) and FTO gene polymorphism (β = 0.14, *p* = 0.04). The variables had the greatest impact on the prediction of the dependent variable (respectively, ΔR2 = 0.15, *p* < 0.001, ΔR2 = 0.064, *p* = 0.001, ΔR2 = 0.055, *p* < 0.01, ΔR2 = 0.018, *p* = 0.04) (tab. 12).

The boys' BMI scores were predicted based on 7 variables: paternal BMI, maternal age at the child’s birth, maternal BMI in pregnancy, experience of prenatal stress, eating habits, maternal education, and MC4R gene polymorphism. The model explained 14.6% of the variance in the dependent variable (*p* < 0.001). Paternal BMI, maternal age at the child’s birth, and maternal BMI before pregnancy had the greatest impact (ΔR2 = 0.053, *p* = 0.002; ΔR2 = 0.027, *p* = 0.03, ΔR2 = 0.022, *p* = 0.02, respectively). Paternal BMI (β = 0.08, *p* = 0.002) and maternal BMI before pregnancy (β = 0.07, *p* = 0.02) were positively associated with the dependent variable, while maternal age at the child’s birth was negatively associated with the dependent variable (β = -0.04, *p* = 0.03) (Table 13).

**Table 13:** Multiple forward regression results predicting BMI z scores, FMI z scores, FFMI z scores and FatM z scores in boys

Variable	R2	β	F	*P*-value
Model 1: BMI z scores	0.146		4.15	< 0.001
Paternal BMI		0.08		0.002
Mother's age at the child’s birth		-0.04		0.03
Maternal BMI before pregnancy		0.07		0.02
Prenatal stress (ref. 0)		0.34		0.09
Eating behaviours (ref. good)		0.28		0.12
Maternal educational level (ref. higher)		0.26		0.19
MC4R (ref. TT)		0.21		0.27
Model 2: FMI z scores	0.080		5.04	< 0.002
Maternal educational level (ref. higher)		0.17		0.02
Mother's age at the child’s birth		-0.15		0.04
Paternal BMI		0.15		0.046
Model 3: FFMI z scores	0.165		5.69	< 0.001
Paternal BMI		0.24		0.001
Eating behaviours (ref. good)		0.16		0.02
Maternal current BMI		0.17		0.02
Mother's age at the child’s birth		-0.14		0.05
Prenatal stress (ref. 0)		0.10		0.14
Paternal educational level (ref. higher)		0.08		0.27
Model 4: FatM z scores	0.085		4.08	< 0.001
Maternal educational level (ref. higher)		0.16		0.04
Paternal BMI		0.15		0.04
Mother's age at the child’s birth		-0.16		0.03
Highest body weight in pregnancy		0.08		0.30

In the group of boys, the variables that remained in the multiple regression model explaining 8% (F = 5.04, *p* < 0.002) of the variability of FMI z scores were maternal education, maternal age at the child’s birth, and paternal BMI (ΔR2 = 0.40, *p* = 0.02, ΔR2 = 0.019, *p* = 0.04, ΔR2 = 0.021, *p* = 0.046, respectively). Paternal BMI (β = 0.17, *p* = 0.02) and maternal education (β = 0.15, *p* = 0.046) were positively associated with FMI z scores, while maternal age at the child’s birth was negatively associated with FMI z scores (β = -0.15, *p* = 0.04) (Table 13).

The stepwise regression model for FFMI z scores in the boys’ group included paternal BMI, eating behavior, current maternal BMI, maternal age at the child’s birth, prenatal stress experience, and paternal education, which explained 16.5% of the dependent variable’s variance. FFMI z scores were positively related to paternal BMI (β = 0.24, *p* < 0.001), eating behaviour (β = 0.16, *p* = 0.02), and current maternal BMI (β = 0.17, *p* = 0.02). These variables had the greatest impact on the prediction of the explained variable (ΔR2 = 0.71, *p* < 0.001, ΔR2 = 0.028, *p* = 0.02, ΔR2 = 0.024, *p* = 0.02, respectively) (Table 13).

Eight point five percent (8.5%) of the variance in FatM scores in the boys’ group was explained by maternal education, paternal BMI, maternal age at the child’s birth, and highest weight during pregnancy. Maternal education (β = 0.16, *p* = 0.04) and paternal BMI (β = 0.15, *p* = 0.04) were positively associated with FatM z scores, and maternal age at the child’s birth was negatively associated with FatM z scores (β = -0.16, *p* = 0.03). Maternal education, paternal BMI and maternal age at the child’s birth had the greatest impact on the prediction of the dependent variable (ΔR2 = 0.037, *p* = 0.04; ΔR2 = 0.019, *p* = 0.04, ΔR2 = 0.023, *p* = 0.03, respectively) (Table 13).

### The effects of ACEs and genetic interactions on body composition parameters

**Fig. 1:**
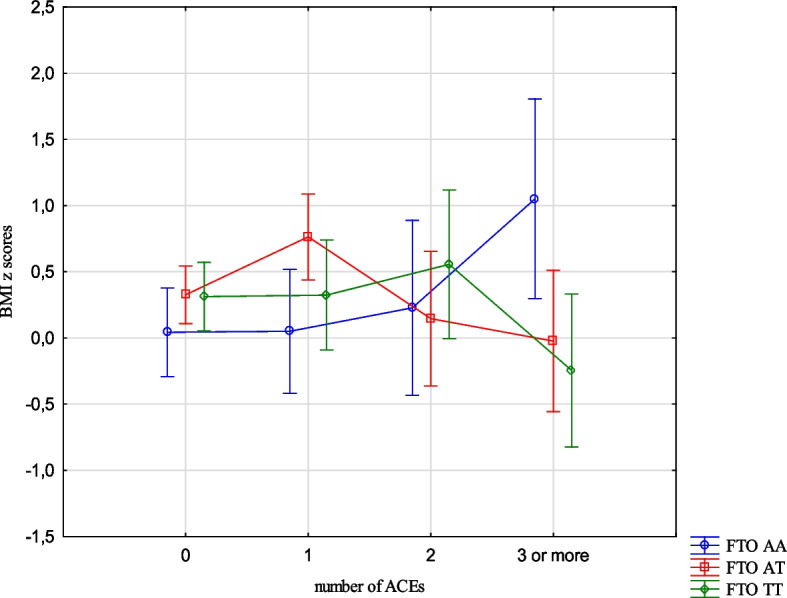
Effects of interaction between FTO polymorphism and ACEs on the BMI z scores

**Fig. 2:**
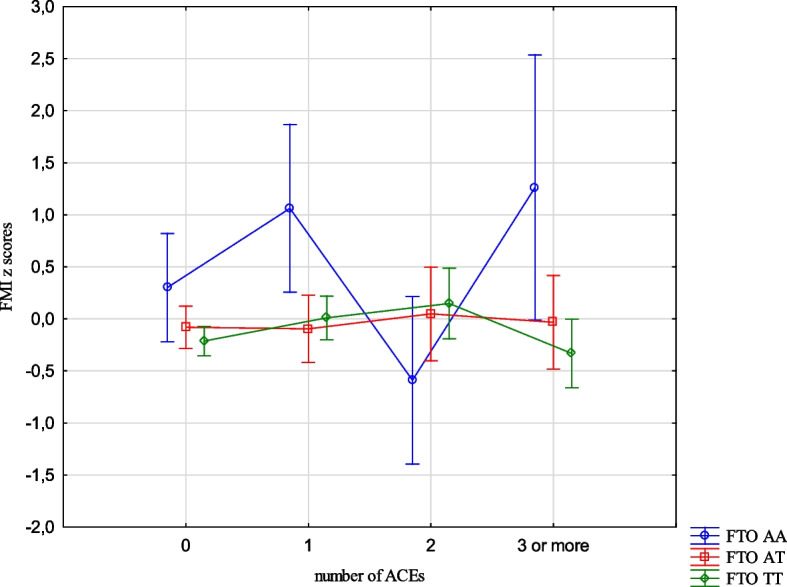
Effects of interaction between MC4R polymorphism and ACEs on FMI z scores

**Fig. 3:**
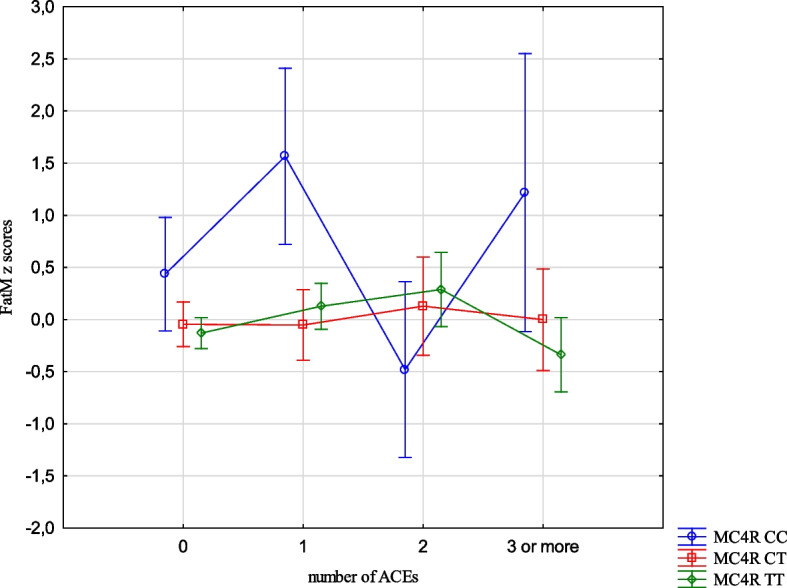
Effects of interaction between MC4R polymorphism and ACEs on FatM z scores

**Fig. 4:**
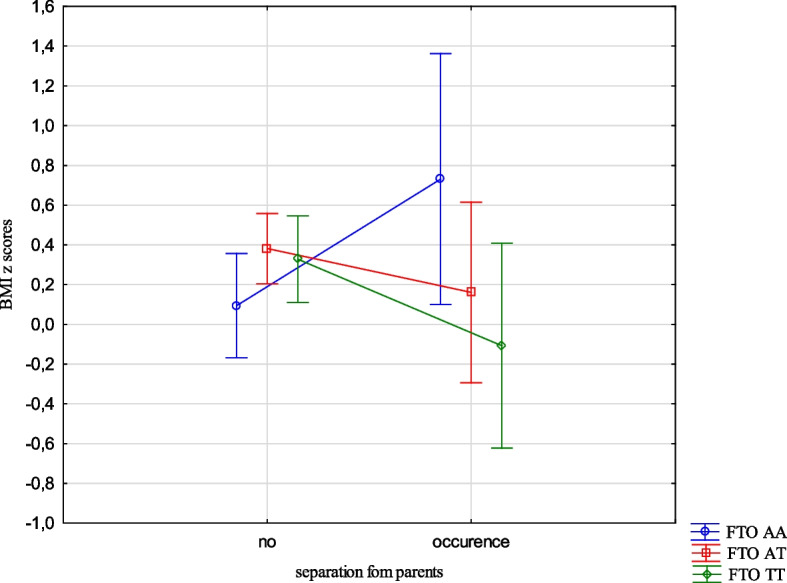
Effects of interaction between FTO polymorphism and separation from parents on BMI z scores

**Fig. 5:**
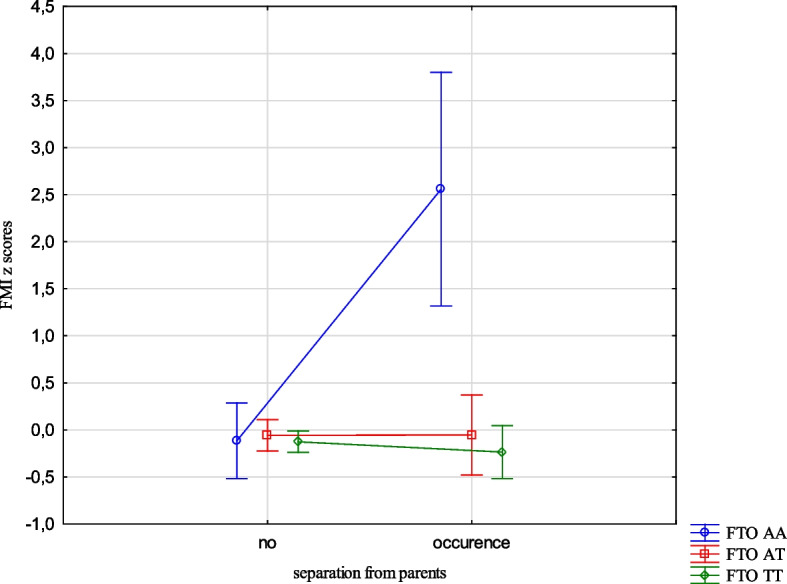
Effects of interaction between MC4R polymorphism and separation from parents on FMI z scores

**Fig. 6:**
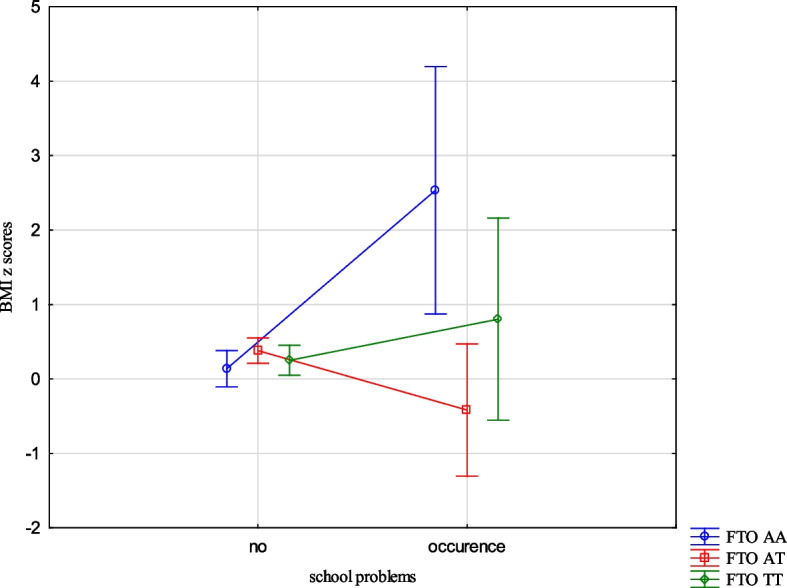
Effects of interaction between FTO polymorphism and school problems on BMI z scores

**Fig. 7:**
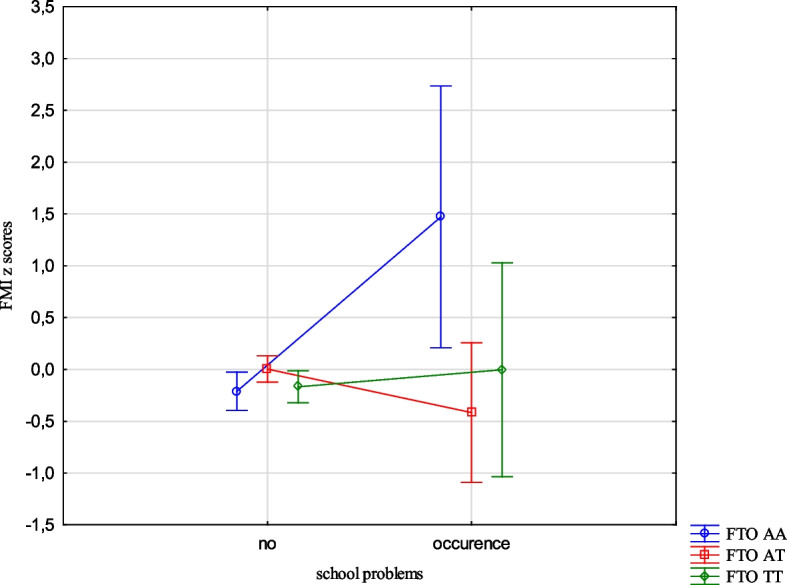
Effects of interaction between FTO polymorphism and school problems on FMI z scores

**Fig. 8:**
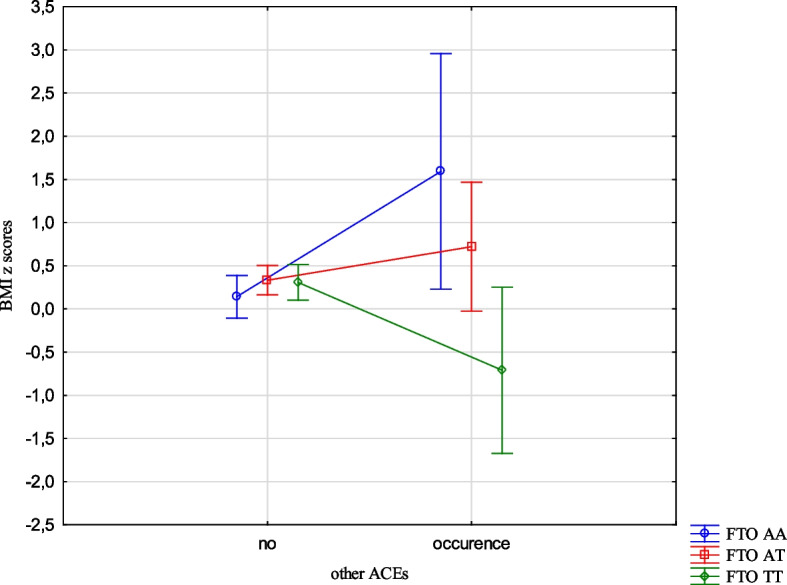
Effects of interaction between FTO polymorphism and other stressful events on BMI z scores

**Fig. 9:**
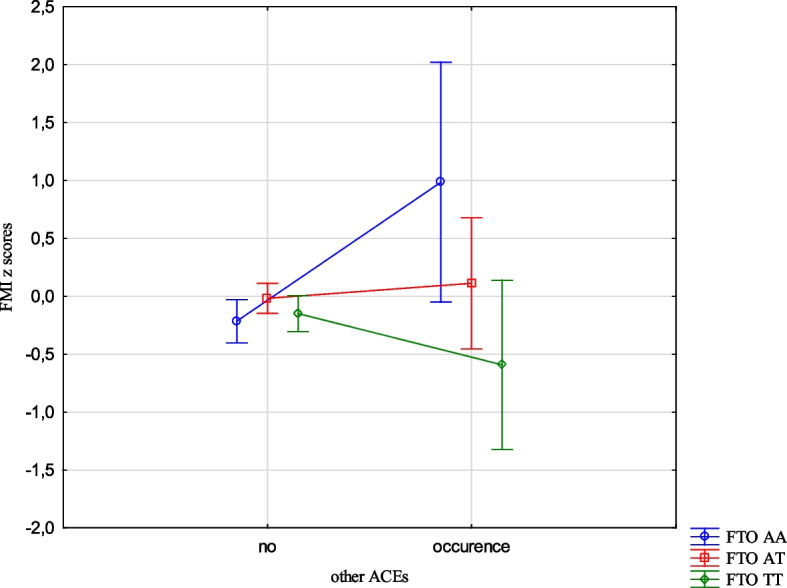
Effects of interaction between FTO polymorphism and other stressful events on FMI z scores

The analysis revealed the effects of the interaction between the FTO and MC4R gene polymorphisms and ACE types on the BMI, FMI, FFMI and FatM z scores. The experience of 3 or more stressors was related to an increased BMI z scores (1.05 vs -0.24, Cohen’s d = 1.05) (Table 14, Fig. 1) in the children with FTO AA compared to TT, and increased FMI and FatM z scores in the children with MC4R CC compared to TT (1.26 vs -0.33, Cohen’s d = 3.20; 0.56 vs -0.36, Cohen’s d = 2.54, respectively). (Table 14, Figs. 2 and 3). Separation from the parents was related to an increase in BMI z scores in the children with FTO AA compared to TT (0.73 vs -0.11, Cohen’s d = 0.62) (Table 14, Fig. 4) and MC4R CC compared to TT. It was also connected with an increase in FMI z scores (2.55 vs -0.23, Cohen’s d = 1.72) and FatM z scores (2.43 vs 0.27, Cohen’s d = 1.39) (Table 14, Fig. 5) in the children with MC4R CC compared to TT. Difficulties at school were related to increased BMI and FMI z scores in the children with FTO AA compared to TT (2.54 vs 0.80, Cohen’s d = 1.90; 1.47 vs -0.003, Cohen’s d = 2.33, respectively) (Table 14, Figs. 6 and 7). The experience of other unspecified stressors was related to higher BMI and FMI z scores in the children with FTO AA compared to TT (1.59 vs -0.71, Cohen’s d = 1.43; 0.98 vs -0.59, Cohen’s d = 1.60, respectively) (Table 14, Figs. 8 and 9).

**Table 14:** Interactions between ACEs and polymorphisms of FTO and MC4R genes

	FTO		MC4R	
	F	p	F	p
BMI z scores				
ACE 0,1,2,3 +	**2.59**	**0.02**	1.80	0.10
Life threatened	2.20	0.11	0.10	0.90
Life threatened witness	1.73	0.17	0.30	0.74
Violence victim	0.58	0.56	0.16	0.85
Violence witness	0.50	0.61	0.22	0.80
Death of someone close	1.65	0.19	0.10	0.91
Family conflicts	1.94	0.15	0.02	0.98
Separation from parent	**3.06**	**0.048**	**3.07**	**0.03**
School problems	**5.73**	**0.004**	1.33	0.27
Other unspecified stressors	**4.60**	**0.01**	0.14	0.87
ACE at least one	0.58	0.55	0.06	0.94
FMI z scores				
ACE 0,1,2,3 +	1.65	0.13	**2.31**	**0.03**
Life threatened	1.32	0.26	0.08	0.92
Life threatened witness	1.96	0.14	0.73	0.48
Violence victim	0.44	0.65	0.11	0.90
Violence witness	0.27	0.76	0.07	0.93
Death of someone close	0.15	0.86	0.55	0.58
Family conflicts	1.55	0.21	0.55	0.58
Separation from parent	0.93	0.40	**8.34**	** < 0.001**
School problems	**4.09**	**0.02**	0.81	0.44
Other unspecified stressors	**3.13**	**0.04**	1.02	0.36
ACE at least one	0.63	0.53	0.26	0.77
FFMI z scores				
ACE 0,1,2,3 +	1.70	0.12	1.65	0.13
Life threatened	2.67	0.07	0.18	0.84
Life threatened witness	0.50	0.61	0.39	0.67
Violence victim	1.19	0.30	0.69	0.50
Violence witness	0.01	0.99	0.74	0.48
Death of someone close	2.33	0.10	0.60	0.55
Family conflicts	2.07	0.13	0.68	0.51
Separation from parent	**3.27**	**0.04**	2.16	0.11
School problems	**4.95**	**0.007**	1.91	0.15
Other unspecified stressors	**3.03**	**0.04**	0.54	0.58
ACE at least one	0.66	0.52	1.27	0.28
FatM z scores				
ACE 0,1,2,3 +	0.80	0.57	**2.27**	**0.04**
Life threatened	0.98	0.38	0.13	0.88
Life threatened witness	0.62	0.54	0.27	0.77
Violence victim	0.41	0.67	0.007	0.99
Violence witness	0.22	0.81	0.13	0.88
Death of someone close	0.31	0.74	0.13	0.88
Family conflicts	1.34	0.26	0.11	0.90
Separation from parent	0.75	0.47	**9.73**	** < 0.001**
School problems	2.28	0.10	0.34	0.71
Other unspecified stressors	1.99	0.14	0.22	0.80
ACE at least one	0.64	0.53	0.07	0.93

*BMI* Body Mass Index, *FMI* Fat Mass Index, *FFMI* Fat Free Mass Index, *FatM* Fat Mass in kg

## Discussion

In the context of previous research, the interactions between genes and the environment seem to be particularly interesting. One less explored aspect pertains to the interplay between unfavourable life experiences and polymorphisms of the FTO rs9939609 and MC4R rs17782313 genes and their impact on body composition parameters. The results of the Copenhagen General Population Study indicate a positive relationship between BMI and WHR (Waist to Hip Ratio) and distress in adults. However, while examining the same relationships using adiposity related genotypes (FTO rs9939609 and MC4R rs17782313) as instrumental variables inverse associations were observed [47]. Overweight is influenced by both genetic and environmental factors. In our study we investigated the association of polymorphisms of the FTO rs9939609 and MC4R rs1778231 genes, which have been well-documented to be related to a higher prevalence of overweight and obesity [2, 5].

The results of our study reveal that environmental factors have a significant influence on body composition changes in school-aged children, while genetic factors show less significance in the multiple regression model. This indicates that environmental factors play a more substantial role in shaping changes in body composition.

The study confirms that the most strongly influential factors on body composition parameters were the paternal BMI, maternal education, highest body weight in pregnancy and mother’s age at the child’s birth, although they explain a low variability of the dependent variable. The limited explanatory capacity of these variables needs further invastigation and additional research. It is essential to acknowledge that beyond the factors explored in this study, numerous others may potentially influence body composition parameters. The available literature provides evidence that children of parents with overweight/obesity are more prone to developing obesity [48–51]. Other studies have indicated a stronger maternal intergenerational relationship with the child’s body mass [48, 49, 52]. However, our findings demonstrate that the paternal BMI has the strongest influence on the child’s body composition parameters. These results align with a meta-analysis that shows a connection between paternal BMI and the child’s weight and/or body fat [13]. The higher the father's BMI, the greater the child's body weight and/or the higher the body fat percentage [53]. Our futher analysis reveals that paternal BMI in addition to BMI z scores significantly affects all other body composition parameters of the child, including FMI, FFMI, and FatM. A possible explanation for the influence of paternal BMI on the children's body composition could be the process of learning by imitation. Parents' weight is frequently related to their lifestyle, and it is well-documented that the dietary habits of individuals with a normal body weight differ from those who have overweight or obesity [54, 55]. Children, being keen observers, often emulate the dietary choices [56] and leisure activities [57] of their parents.

A high pregnancy weight is positively correlated with BMI z scores and FatM z scores. Previous research demonstrated that maternal gestational weight gain is associated with the child’s obesity and high waist circumference [58]. Maternal overnutrition may affect the development of adipocytes and their capacity to regulate the appetite control system and energy metabolism later in life [59], which might lead to increased body fat in the offspring.

The mothers without a university education had children with higher FMI z scores and FatM z scores. There is an inverse relationship between the parents’ level of education and obesity of their children, with the lowest level of education corresponding to the highest prevalence of obesity in the children. The parents’education level has a significant impact on the child’s body composition [28]. Education is also related to other components of socioeconomic status. Individuals with lower education tend to have lower income, which affects the quality of the food they choose, including the food served to their children [60]. Moreover, mothers with lower education levels are less likely to breastfeed their children [61], which is also a protective factor against the child’s overweight and obesity later in life [62]. The socioeconomic conditions of parents are very important in shaping the children's eating habits since they influence the type of food available at home [63]. Studies indicate that the diet of children whose parents declared low socioeconomic status exhibit a higher consumption of high-energy food with low nutritional value [64] and a higher consumption of sugary drinks [65] at a preschool age. The mother's impact on the child's nutritional preferences is already present in fetal life, as her diet during pregnancy and breastfeeding shapes the future nutritional preferences of her child [66, 67].

The presence of risk alleles of the FTO and MC4R genes was included in the models describing the variability of the body composition parameters, but the relationships with MC4R were not statistically significant. However, we were interested in how genetic susceptibility and the coexistence of unfavourable environmental factors, such as ACEs would affect the body composition parameters. Experiencing three stressors while being homozygous for the risk allele was associated with higher BMI z scores for the FTO gene and higher FMI z scores and FatM z scores for the MC4R gene compared to the children who did not carry the risk allele. The results indicate interactions between genetic and environmental factors. The presence of risk alleles alone is not a factor for the occurrence of changes in children's weight and body composition, but it may become one depending on the environment. The results of our study indicate that the presence of 3 or more unfavourable life experiences could potentially serve as a contributing factor. Upon separate examination of different types of stressors, such as separation from parents, problems at school and other unspecified types of stressors significant associations emerged.The children who were separated from their parents and also carried the risk homozygous alleles of the FTO and MC4R genes exhibited higher BMI z scores, FMI z scores, and FatM z scores. Similarly, those who were homozygous for the risk allele of the FTO gene and had serious problems in school showed higher BMI z scores, FMI z scores, and FFMI z scores. The occurrence of other unspecified stressful situations, combined with being homozygous for the risk allele of the FTO gene was also related to higher BMI z scores, FMI z scores and FFMI z scores. While considering only life stressors as the influencing factor no significant correlation was found with the body composition parameters in the children. The presence of the risk allele of the FTO gene did not demonstrate an important impact on the body composition parameters, whereas the presence of the risk allele of the MC4R gene was associated with increased FMI z scores and FFMI z scores. However, when the children were carriers of the risk allele as well as experienced ACEs, there was a statistically significant effect on all body composition parameters.

The gene polymorphisms investigated in our study were selected for analysis based on strong evidence supporting their association with BMI and body weight in children [2, 5]. It is worth emphasizing, however, that no studies have so far demonstrated a direct association between the FTO gene polymorphism and a resting metabolic rate [4]. The development of excess body weight in children with the unfavourable allele of the FTO gene is attributed to their tendency to consume meals that are higher in energy and fat content [68]. Similarly for the MC4R gene, the presence of the C allele leads to increased eating pleasure, reduced satiety, and a tendency to eat when not hungry, which may contribute to obesity [5]. Thus the mere presence of an unfavourable allele of the gene does not singularly determine changes in the body weight. However, if environmental factors, such as ACEs, which may also affect food choices [69] co-occure, the risk of changes in body composition parameters increases.

## Conclusions

The results of our study indicate that within the multiple regression model, genetic factors exhibit a lower level of significance compared to the environment when elucidating alterations in body composition parameters among children. The presence of the risk allele does not determine a decisive influence on changes in body composition. However, together with the simultaneous occurrence of unfavourable environmental factors, such as ACEs a discernible interaction effect emerges, leading to an increase in BMI z scores and FMI z scores in children.

## Strengths and weaknesses of the study

The strength of our study lies in the use of variables related to body weight normality, which include not only weight itself and the BMI derived from it but also indicators based on fat mass content (FMI) and fat-free mass content (FFMI). Another significant aspect is the comprehensive consideration of numerous environmental, perinatal, ACE and lifestyle factors as well as the FTO and MC4R gene polymorphisms to examine their relationships with the body composition parameters. The influence of environmental factors on the risk of overweight and obesity in children has been extensively studied; however, research on ACEs and their association with body composition remains limited. To our knowledge, there have been only two previously published studies [28, 29] that focused entirely on the relationship between ACEs and the body composition parameters, and their results were contradictory. Our study, therefore, represents an attempt to further explain this phenomenon. Another strength of the investigation is the examination of interactions between the genes and the environment and their mutual influence on the body composition parameters.

A weakness of the study is the use of BMI as a tool to assess underweight, overweight, and obesity in children. Based on existing research, the use of BMI may not be an appropriate method for such an assessment since it does not consider the content of body fat and fat-free mass [70]. While assessing body weight based on BMI, there is a possibility of overestimating the risk of excessive weight gain in children with a high amount of lean muscle mass [71, 72]. On the other hand, it is possible to underestimate the risk in children who, despite their relatively low body weight, have a high fat content hazardous to health [73]. A weakness of the study is our reliance on information about the parents' weight and height derived solely from the questionnaires. This may be associated with an overestimation of height (especially in the case of men) and an underestimation of body weight (in women and individuals who are overweight or obese) [74]. We did not check food intake, physical activity and time of last urination prior to the body composition analysis. However, we would like to point out that other studies on this subject indicate that differences in body composition depending on external factors are not clinically significant [75]. Furthermore, limitations include the retrospective study of stressors, which increases the risk of memory error.

### Supplementary Information

**Additional file 1.** Correlations between BMI z scores, FMI z scores, FFMI z scores, FatM z scores and covariates. Prevalence of underweight, overweight and obesity diagnosed according to IOTF (Body weight status) and according to McCarthy criteria (Body fat status), and association between body composition parameters and parental, environmental and genetic factors – statistically significant results. Interactions between ACEs and polymorphisms of FTO and MC4R genes.

## Data Availability

Due to the sensitive nature of the data supporting the conclusions of the article, only selective access to data is offered on reasonable request to the principal investigator (MD-W).
