# Supplementary material for: FTO and MC4R polymorphisms, and selected pre-, peri- and postnatal factors as determinants of body mass index and fatness in children: a thorough analysis of the associations
Source: J Physiol Anthropol. 2023 Dec 8;42:29. doi: 10.1186/s40101-023-00344-1 (PMC10704801; doi:10.1186/s40101-023-00344-1)
Supplement: Supplementary file 1 — Additional file 1. Correlations between BMI z scores, FMI z scores, FFMI z scores, FatM z scores and covariates. Prevalence of underweight, overweight and obesity diagnosed according to IOTF (Body weight status) and according to McCarthy criteria (Body fat status), and association between body composition parameters and parental, environmental and genetic factors – statistically significant results. Interactions between ACEs and polymorphisms of FTO and MC4R genes. [file 40101_2023_344_MOESM1_ESM.docx]

Correlations between BMI z scores, FMI z scores, FFMI z scores, FatM z scores and covariates

|  |  | 1 | 2 | 3 | 4 | 5 | 6 | 7 | 8 | 9 | 10 |
| --- | --- | --- | --- | --- | --- | --- | --- | --- | --- | --- | --- |
| 1 | BMI z scores | - |  |  |  |  |  |  |  |  |  |
| 2 | FMI z scores | 0.86* | - |  |  |  |  |  |  |  |  |
| 3 | FFMI z scores | 0.84* | 0.60* | - |  |  |  |  |  |  |  |
| 4 | FatM z scores | 0.85* | 0.97* | 0.61* | - |  |  |  |  |  |  |
| 5 | highest body weight in pregnancy | 0.21* | 0.19* | 0.16* | 0.21* | - |  |  |  |  |  |
| 6 | pregnancy duration | -0.04 | -0.05 | -0.04 | -0.03 | 0.02 | - |  |  |  |  |
| 7 | maternal age at the child’s birth | -0.14* | -0.14* | -0.13* | -0.14* | -0.01 | -0.09 | - |  |  |  |
| 8 | maternal current BMI | 0.18* | 0.13* | 0.16* | 0.13* | 0.66* | -0.02 | 0.06 | - |  |  |
| 9 | maternal BMI before pregnancy | 0.18* | 0.13* | 0.15* | 0.12* | 0.73* | <0.01 | 0.07 | 0.84* | - |  |
| 10 | paternal current BMI | 0.30* | 0.24* | 0.27* | 0.22* | 0.14* | -0.06 | -0.02 | 0.12* | 0.04 | - |

### Prevalence of underweight, overweight and obesity diagnosed according to IOTF (Body weight status) and according to McCarthy criteria (Body fat status), and association between body composition parameters and parental, environmental and genetic factors – statistically significant results

| Variable | | | Body weight status | | | | | Body fat status | | | |  | BMI z scores | | FMI z scores | | FFMI z scores | | FatM z scores | |
| --- | --- | --- | --- | --- | --- | --- | --- | --- | --- | --- | --- | --- | --- | --- | --- | --- | --- | --- | --- | --- |
|  | n |  | | Underweight | Proper weight | Overweight | Obesity | Underweight | Proper weight | Overweight | Obesity |  | $\bar{x}$ | SD | $\bar{x}$ | SD | $\bar{x}$ | SD | $\bar{x}$ | SD |
|  |  |  | |  |  |  |  |  |  |  |  |  |  |  |  |  |  |  |  |  |
| Maternal BMI before pregnancy | 426 |  | |  |  |  |  |  |  |  |  |  |  |  |  |  |  |  |  |  |
| Underweight |  |  | | 10 | 22 | 7 | 0 | 1 | 27 | 5 | 6 |  | -0.06 | 1.38 | -0.19 | 0.90 |  |  | -0.16 | 0.96 |
|  |  |  | | 2.35% | 5.16% | 1.64% | 0,00% | 0.23% | 6.34% | 1.17% | 1.41% |  |  |  |  |  |  |  |  |  |
| Proper weight |  |  | | 33 | 237 | 44 | 15 | 0 | 237 | 51 | 43 |  | 0.27 | 1.19 | -0.14 | 0.89 |  |  | -0.06 | 0.91 |
|  |  |  | | 7.75% | 55.63% | 10.33% | 3.52% | 0,00% | 55.63% | 11.97% | 10.09% |  |  |  |  |  |  |  |  |  |
| Overweight |  |  | | 1 | 35 | 6 | 5 | 0 | 31 | 8 | 6 |  | 0.59 | 1.04 | 0.11 | 1.06 |  |  | 0.17 | 1.15 |
|  |  |  | | 0.23% | 8.22% | 1.41% | 1.17% | 0,00% | 7.28% | 1.88% | 1.41% |  |  |  |  |  |  |  |  |  |
| Obesity |  |  | | 0 | 4 | 6 | 1 | 0 | 3 | 4 | 4 |  | 1.35 | 1.18 | 0.59 | 0.87 |  |  | 0.66 | 0.84 |
|  |  |  | | 0,00% | 0.94% | 1.41% | 0.23% | 0,00% | 0.7% | 0.94% | 0.94% |  |  |  |  |  |  |  |  |  |
|  |  | χ^2^ | | **34.32** |  |  |  | **20.47** |  |  |  | F | **5.11** |  | **3.28** |  |  |  | **3.07** |  |
|  |  | P | | **<0.001** |  |  |  | **0.01** |  |  |  | p | **<0.001** |  | **0.02** |  |  |  | **0.03** |  |
|  |  |  | |  |  |  |  |  |  |  |  |  | underweight vs obesity, proper weight vs obesity^#^ |  | proper weight vs obesity^#^ |  |  |  | no differences^#^ |  |
| Maternal current BMI | 431 |  | |  |  |  |  |  |  |  |  |  |  |  |  |  |  |  |  |  |
| Underweight |  |  | | 4 | 6 | 4 | 0 |  |  |  |  |  | 0.03 | 1.51 | -0.16 | 1.19 | -0.03 | 0.90 | -0.02 | 1.26 |
|  |  |  | | 0.93% | 1.39% | 0.93% | 0,00% |  |  |  |  |  |  |  |  |  |  |  |  |  |
| Proper weight |  |  | | 35 | 218 | 34 | 10 |  |  |  |  |  | 0.17 | 1.16 | -0.18 | 0.85 | -0.04 | 0.82 | -0.12 | 0.86 |
|  |  |  | | 8.12% | 50.58% | 7.89% | 2.32% |  |  |  |  |  |  |  |  |  |  |  |  |  |
| Overweight |  |  | | 4 | 60 | 16 | 8 |  |  |  |  |  | 0.61 | 1.21 | 0.09 | 1.03 | 0.36 | 0.98 | 0.19 | 1.12 |
|  |  |  | | 0.93% | 13.92% | 3.71% | 1.86% |  |  |  |  |  |  |  |  |  |  |  |  |  |
| Obesity |  |  | | 1 | 16 | 11 | 4 |  |  |  |  |  | 0.92 | 1.26 | 0.34 | 1.03 | 0.43 | 0.96 | 0.42 | 1.10 |
|  |  |  | | 0.23% | 3.71% | 2.55% | 0.93% |  |  |  |  |  |  |  |  |  |  |  |  |  |
|  |  | χ^2^ | | **34.92** |  |  |  |  |  |  |  | F | **6,23** |  | **4.48** |  | **6.68** |  | **4,83** |  |
|  |  | P | | **<0.001** |  |  |  |  |  |  |  | p | **<0,001** |  | **0.004** |  | **<0.001** |  | **0.002** |  |
|  |  |  | |  |  |  |  |  |  |  |  |  | proper weight vs overweight,  proper weight vs obesity^#^ |  | proper weight vs obesity^#^ |  | proper weight vs overweight  proper weight vs obesity^#^ |  | proper weight vs overweight,  proper weight vs obesity^#^ |  |
| Paternal current BMI | 408 |  | |  |  |  |  |  |  |  |  |  |  |  |  |  |  |  |  |  |
| Proper weight |  |  | | 16 | 97 | 15 | 3 |  |  |  |  |  | 0.02 | 1.09 | -0.30 | 0.83 | -0.09 | 0.79 | -0.21 | 0.89 |
|  |  |  | | 3.93% | 23.83% | 3.69% | 0.74% |  |  |  |  |  |  |  |  |  |  |  |  |  |
| Overweight |  |  | | 22 | 137 | 33 | 5 |  |  |  |  |  | 0.25 | 1.16 | -0.14 | 0.77 | 0.04 | 0.87 | -0.07 | 0.81 |
|  |  |  | | 5.41% | 33.66% | 8.11% | 1.23% |  |  |  |  |  |  |  |  |  |  |  |  |  |
| Obesity |  |  | | 1 | 52 | 15 | 11 |  |  |  |  |  | 0.99 | 1.13 | 0.35 | 1.15 | 0.51 | 0.79 | 0.43 | 1.17 |
|  |  |  | | 0.25% | 12.78% | 3.69% | 2.7% |  |  |  |  |  |  |  |  |  |  |  |  |  |
|  |  | χ^2^ | | **27.88** |  |  |  |  |  |  |  | F | **13.44** |  | **9.48** |  | **10.36** |  | **8.50** |  |
|  |  | P | | **<0.001** |  |  |  |  |  |  |  | p | **<0.001** |  | **<0.001** |  | **<0.001** |  | **<0.001** |  |
|  |  |  | |  |  |  |  |  |  |  |  |  | proper weight vs obesity, overweight vs obesity^#^ |  | proper weight vs obesity, overweight vs obesity^#^ |  | proper weight vs obesity, overweight vs obesity^#^ |  | proper weight vs obesity, overweight vs obesity^#^ |  |
| Maternal education level | 439 |  | |  |  |  |  |  |  |  |  |  |  |  |  |  |  |  |  |  |
| Primary |  |  | | 0 | 4 | 0 | 1 | 0 | 3 | 1 | 1 |  | 0.73 | 1.37 | 0.06 | 1.11 |  |  | 0.16 | 1.19 |
|  |  |  | | 0,00% | 0.91% | 0,00% | 0.23% | 0,00% | 0.68% | 0.23% | 0.23% |  |  |  |  |  |  |  |  |  |
| Vocational |  |  | | 3 | 26 | 9 | 8 | 0 | 25 | 8 | 13 |  | 0.78 | 1.49 | 0.47 | 1.36 |  |  | 0.52 | 1.41 |
|  |  |  | | 0.68% | 5.92% | 2.05% | 1.82% | 0,00% | 5.69% | 1.82% | 2.96% |  |  |  |  |  |  |  |  |  |
| Secondary |  |  | | 15 | 70 | 18 | 8 | 1 | 73 | 14 | 22 |  | 0.37 | 1.39 | 0.02 | 1.03 |  |  | 0.09 | 1.09 |
|  |  |  | | 3.42% | 15.95% | 4.1% | 1.82% | 0.23% | 16.63% | 3.19% | 5.01% |  |  |  |  |  |  |  |  |  |
| University |  |  | | 26 | 205 | 41 | 5 | 0 | 204 | 48 | 26 |  | 0.20 | 1.06 | -0.23 | 0.73 |  |  | -0.15 | 0.76 |
|  |  |  | | 5.92% | 46.7% | 9.34% | 1.14% | 0,00% | 46.47% | 10.93% | 5.92% |  |  |  |  |  |  |  |  |  |
|  |  | χ^2^ | | **29.64** |  |  |  | **20.24** |  |  |  | F | **3.37** |  | **8.72** |  |  |  | **7.20** |  |
|  |  | P | | **0.001** |  |  |  | **0.02** |  |  |  | p | **0.02** |  | **<0.001** |  |  |  | **<0.001** |  |
|  |  |  | |  |  |  |  |  |  |  |  |  | secondary vs higher^#^ |  | secondary vs vocational, secondary vs higher^#^ |  |  |  | Secondary vs vocational, secondary vs higher |  |
| Paternal education level | 409 |  | |  |  |  |  |  |  |  |  |  |  |  |  |  |  |  |  |  |
| Primary |  |  | | 11 | 7 | 2 | 1 |  |  |  |  |  | 0.53 | 1.39 | 0.18 | 1.19 |  |  | 0.20 | 1.18 |
|  |  |  | | 0.24% | 1.65% | 0.47% | 0.24% |  |  |  |  |  |  |  |  |  |  |  |  |  |
| Vocational |  |  | | 8 | 49 | 23 | 6 |  |  |  |  |  | 0.60 | 1.32 | 0.15 | 1.00 |  |  | 0.25 | 1.08 |
|  |  |  | | 1.89% | 11.58% | 5.44% | 1.42% |  |  |  |  |  |  |  |  |  |  |  |  |  |
| Secondary |  |  | | 11 | 88 | 17 | 9 |  |  |  |  |  | 0.40 | 1.24 | -0.01 | 1.04 |  |  | 0.05 | 1.05 |
|  |  |  | | 2.6% | 20.8% | 4.02% | 2.13% |  |  |  |  |  |  |  |  |  |  |  |  |  |
| University |  |  | | 19 | 154 | 24 | 4 |  |  |  |  |  | 0.15 | 1.04 | -0.26 | 0.71 |  |  | -0.17 | 0.74 |
|  |  |  | | 4.49% | 36.41% | 5.67% | 0.95% |  |  |  |  |  |  |  |  |  |  |  |  |  |
|  |  | χ^2^ | | **18.51** |  |  |  |  |  |  |  | F | **3.38** |  | **5.21** |  |  |  | **4.60** |  |
|  |  | P | | **0.03** |  |  |  |  |  |  |  | p | **0.02** |  | **0.002** |  |  |  | **0.004** |  |
|  |  |  | |  |  |  |  |  |  |  |  |  |  |  |  |  |  |  |  |  |
| Physical activity | 454 |  | |  |  |  |  |  |  |  |  |  |  |  |  |  |  |  |  |  |
| Bad |  |  | | 42 | 242 | 58 | 17 |  |  |  |  |  |  |  |  |  |  |  |  |  |
|  |  |  | | 9.25% | 53.3% | 12.78% | 3.74% |  |  |  |  |  |  |  |  |  |  |  |  |  |
| Good |  |  | | 3 | 73 | 12 | 7 |  |  |  |  |  |  |  |  |  |  |  |  |  |
|  |  |  | | 0.66% | 16.08% | 2.64% | 1.54% |  |  |  |  |  |  |  |  |  |  |  |  |  |
|  |  | χ^2^ | | **8.08** |  |  |  |  |  |  |  |  |  |  |  |  |  |  |  |  |
|  |  | P | | **0.04** |  |  |  |  |  |  |  |  |  |  |  |  |  |  |  |  |
| Birthweight | 441 |  | |  |  |  |  |  |  |  |  |  |  |  |  |  |  |  |  |  |
| <2500 |  |  | | 6 | 10 | 2 | 2 |  |  |  |  |  |  |  |  |  |  |  |  |  |
|  |  |  | | 1.36% | 2.27% | 0.45% | 0.45% |  |  |  |  |  |  |  |  |  |  |  |  |  |
| 2500-4000 |  |  | | 36 | 255 | 52 | 18 |  |  |  |  |  |  |  |  |  |  |  |  |  |
|  |  |  | | 8.16% | 57.82% | 11.79% | 4.08% |  |  |  |  |  |  |  |  |  |  |  |  |  |
| >4000 |  |  | | 2 | 42 | 14 | 2 |  |  |  |  |  |  |  |  |  |  |  |  |  |
|  |  |  | | 0.45% | 9.52% | 3.17% | 0.45% |  |  |  |  |  |  |  |  |  |  |  |  |  |
|  |  | χ^2^ | | **16.25** |  |  |  |  |  |  |  |  |  |  |  |  |  |  |  |  |
|  |  | p | | **0.02** |  |  |  |  |  |  |  |  |  |  |  |  |  |  |  |  |
| Weight gain during pregnancy | 434 |  | |  |  |  |  |  |  |  |  |  |  |  |  |  |  |  |  |  |
| Exceeded |  |  | | 2 | 22 | 12 | 5 |  |  |  |  |  |  |  |  |  |  |  |  |  |
|  |  |  | | 0.46% | 5.07% | 2.76% | 1.15% |  |  |  |  |  |  |  |  |  |  |  |  |  |
| Non-exceeded |  |  | | 42 | 279 | 54 | 18 |  |  |  |  |  |  |  |  |  |  |  |  |  |
|  |  |  | | 9.68% | 64.29% | 12.44% | 4.15% |  |  |  |  |  |  |  |  |  |  |  |  |  |
|  |  | χ^2^ | | **12.79** |  |  |  |  |  |  |  |  |  |  |  |  |  |  |  |  |
|  |  | p | | **0.01** |  |  |  |  |  |  |  |  |  |  |  |  |  |  |  |  |
| ACE | 454 |  | |  |  |  |  |  |  |  |  |  |  |  |  |  |  |  |  |  |
| 0 |  |  | | 18 | 189 | 35 | 8 |  |  |  |  |  |  |  |  |  |  |  |  |  |
|  |  |  | | 3.96% | 41.63% | 7.71% | 1.76% |  |  |  |  |  |  |  |  |  |  |  |  |  |
| 1 |  |  | | 13 | 67 | 19 | 10 |  |  |  |  |  |  |  |  |  |  |  |  |  |
|  |  |  | | 2.86% | 14.76% | 4.19% | 2.2% |  |  |  |  |  |  |  |  |  |  |  |  |  |
| 2 |  |  | | 8 | 28 | 8 | 5 |  |  |  |  |  |  |  |  |  |  |  |  |  |
|  |  |  | | 1.76% | 6.17% | 1.76% | 1.1% |  |  |  |  |  |  |  |  |  |  |  |  |  |
| 3+ |  |  | | 6 | 31 | 8 | 1 |  |  |  |  |  |  |  |  |  |  |  |  |  |
|  |  |  | | 1.32% | 6.83% | 1.76% | 0.22% |  |  |  |  |  |  |  |  |  |  |  |  |  |
|  |  | χ^2^ | | **17,27** |  |  |  |  |  |  |  |  |  |  |  |  |  |  |  |  |
|  |  | p | | **0.04** |  |  |  |  |  |  |  |  |  |  |  |  |  |  |  |  |
| Family conflicts | 425 |  | |  |  |  |  |  |  |  |  |  |  |  |  |  |  |  |  |  |
| No |  |  | | 28 | 256 | 54 | 13 | 0 | 252 | 53 | 46 |  |  |  |  |  |  |  |  |  |
|  |  |  | | 6.59% | 60.24% | 12.71% | 3.06% | 0,00% | 59.29% | 12.47% | 10.82% |  |  |  |  |  |  |  |  |  |
| Yes |  |  | | 13 | 42 | 12 | 7 | 1 | 48 | 13 | 12 |  |  |  |  |  |  |  |  |  |
|  |  |  | | 3.06% | 9.88% | 2.82% | 1.65% | 0.24% | 11.29% | 3.06% | 2.82% |  |  |  |  |  |  |  |  |  |
|  |  | χ^2^ | | **12.44** |  |  |  | **5.83** |  |  |  |  |  |  |  |  |  |  |  |  |
|  |  | P | | **0.01** |  |  |  | **0.12** |  |  |  |  |  |  |  |  |  |  |  |  |
| Separation from parents |  |  | |  |  |  |  |  |  |  |  |  |  |  |  |  |  |  |  |  |
| No |  |  | | 30 | 264 | 57 | 16 | 0 | 263 | 57 | 47 |  |  |  |  |  |  |  |  |  |
|  |  |  | | 7.08% | 62.26% | 13.44% | 3.77% | 0,00% | 62.03% | 13.44% | 11.08% |  |  |  |  |  |  |  |  |  |
| Yes |  |  | | 11 | 33 | 9 | 4 | 1 | 37 | 8 | 11 |  |  |  |  |  |  |  |  |  |
|  |  |  | | 2.59% | 7.78% | 2.12% | 0.94% | 0.24% | 8.73% | 1.89% | 2.59% |  |  |  |  |  |  |  |  |  |
|  |  | χ^2^ | | **8.44** |  |  |  | **8.35** |  |  |  |  |  |  |  |  |  |  |  |  |
|  |  | P | | **0.04** |  |  |  | **0.04** |  |  |  |  |  |  |  |  |  |  |  |  |
| Violence witness | 424 |  | |  |  |  |  | 0 | 277 | 61 | 53 |  |  |  |  |  |  |  |  |  |
| No |  |  | |  |  |  |  | 0,00% | 65.33% | 14.39% | 12.5% |  |  |  |  |  |  |  |  |  |
|  |  |  | |  |  |  |  | 1 | 22 | 4 | 6 |  |  |  |  |  |  |  |  |  |
| Yes |  |  | |  |  |  |  | 0.24% | 5.19% | 0.94% | 1.42% |  |  |  |  |  |  |  |  |  |
|  |  | χ^2^ | |  |  |  |  | **12.63** |  |  |  |  |  |  |  |  |  |  |  |  |
|  |  | P | |  |  |  |  | **0.01** |  |  |  |  |  |  |  |  |  |  |  |  |
| Disease/death of someone close | 426 |  | |  |  |  |  |  |  |  |  |  |  |  |  |  |  |  |  |  |
| No |  |  | |  |  |  |  | 0 | 253 | 49 | 49 |  |  |  |  |  |  |  |  |  |
|  |  |  | |  |  |  |  | 0,00% | 59.39% | 11.5% | 11.5% |  |  |  |  |  |  |  |  |  |
| Yes |  |  | |  |  |  |  | 1 | 47 | 17 | 10 |  |  |  |  |  |  |  |  |  |
|  |  | χ^2^ | |  |  |  |  | 0.23% | 11.03% | 3.99% | 2.35% |  |  |  |  |  |  |  |  |  |
|  |  | P | |  |  |  |  | **8.50** |  |  |  |  |  |  |  |  |  |  |  |  |
| School problems | 423 |  | |  |  |  |  | 0 | 293 | 63 | 55 |  |  |  |  |  |  |  |  |  |
| No |  |  | |  |  |  |  | 0,00% | 69.27% | 14.89% | 13,00% |  |  |  |  |  |  |  |  |  |
|  |  |  | |  |  |  |  | 1 | 6 | 2 | 3 |  |  |  |  |  |  |  |  |  |
| Yes |  |  | |  |  |  |  | 0.24% | 1.42% | 0.47% | 0.71% |  |  |  |  |  |  |  |  |  |
|  |  |  | |  |  |  |  | **36.16** |  |  |  |  |  |  |  |  |  |  |  |  |
|  |  |  | |  |  |  |  | **<0.001** |  |  |  |  |  |  |  |  |  |  |  |  |
|  | 454 |  | |  |  |  |  | **0.03** |  |  |  |  |  |  |  |  |  |  |  |  |
| Boys |  |  | |  |  |  |  | 0.22% | 32.60% | 10.57% | 8.81% |  |  |  |  |  |  |  |  |  |
|  |  |  | |  |  |  |  | 0 | 166 | 24 | 27 |  |  |  |  |  |  |  |  |  |
| Girls |  |  | |  |  |  |  | 0.00% | 36.56% | 5.29% | 5.95% |  |  |  |  |  |  |  |  |  |
|  |  | χ^2^ | |  |  |  |  | **11.70** |  |  |  |  |  |  |  |  |  |  |  |  |
|  |  | P | |  |  |  |  | **0.01** |  |  |  |  |  |  |  |  |  |  |  |  |
| At least one disease before pregnancy | 533 |  | |  |  |  |  |  |  |  |  |  |  |  |  |  |  |  |  |  |
| No |  |  | |  |  |  |  | 0 | 237 | 57 | 42 |  |  |  |  |  |  |  |  |  |
|  |  |  | |  |  |  |  | 0,00% | 52.2% | 12.56% | 9.25% |  |  |  |  |  |  |  |  |  |
| Yes |  |  | |  |  |  |  | 1 | 77 | 15 | 25 |  |  |  |  |  |  |  |  |  |
|  |  | χ^2^ | |  |  |  |  | 0.22% | 16.96% | 3.3% | 5.51% |  |  |  |  |  |  |  |  |  |
|  |  | P | |  |  |  |  | **8.66** |  |  |  |  |  |  |  |  |  |  |  |  |
| MC4R |  |  | |  |  |  |  | **0.03** |  |  |  |  |  |  |  |  |  |  |  |  |
| CC |  |  | |  |  |  |  |  |  |  |  |  |  |  | 0.35 | 1.32 |  |  | 0.54 | 1.46 |
|  |  |  | |  |  |  |  |  |  |  |  |  |  |  |  |  |  |  |  |  |
| CT |  |  | |  |  |  |  |  |  |  |  |  |  |  | -0.06 | 0.92 |  |  | -0.02 | 0.92 |
|  |  |  | |  |  |  |  |  |  |  |  |  |  |  |  |  |  |  |  |  |
| TT |  |  | |  |  |  |  |  |  |  |  |  |  |  | -0.13 | 0.90 |  |  | -0.05 | 0.95 |
|  |  |  | |  |  |  |  |  |  |  |  |  |  |  |  |  |  |  |  |  |
|  |  |  | |  |  |  |  |  |  |  |  | F |  |  | **3.11** |  |  |  | **4.09** |  |
|  |  |  | |  |  |  |  |  |  |  |  | p |  |  | **0.04** |  |  |  | **0.02** |  |
|  |  |  | |  |  |  |  |  |  |  |  |  |  |  | CCvsTT# |  |  |  | CC vs CT  CCvs TT# |  |
| Screen time | 454 |  | |  |  |  |  |  |  |  |  |  |  |  |  |  |  |  |  |  |
| >2h |  |  | |  |  |  |  |  |  |  |  |  | 0.42 | 1.25 | 0.01 | 1.02 |  |  | 0.11 | 1.09 |
|  |  |  | |  |  |  |  |  |  |  |  |  |  |  |  |  |  |  |  |  |
| ≤2h |  |  | |  |  |  |  |  |  |  |  |  | 0.18 | 1.17 | -0.20 | 0.81 |  |  | -0.16 | 0.79 |
|  |  |  | |  |  |  |  |  |  |  |  | t | **2.03** |  | **2.44** |  |  |  | **2.92** |  |
|  |  |  | |  |  |  |  |  |  |  |  | P | **0.04** |  | **0.02** |  |  |  | **0.003** |  |
| Type of pregnancy | 437 |  | |  |  |  |  |  |  |  |  |  |  |  |  |  |  |  |  |  |
| Single |  |  | |  |  |  |  |  |  |  |  |  | 0.33 | 1.20 |  |  |  |  | -0.01 | 0.95 |
|  |  |  | |  |  |  |  |  |  |  |  |  |  |  |  |  |  |  |  |  |
| Twin |  |  | |  |  |  |  |  |  |  |  |  | -0.31 | 1.28 |  |  |  |  | -0.43 | 0.72 |
|  |  |  | |  |  |  |  |  |  |  |  |  |  |  |  |  |  |  |  |  |
|  |  |  | |  |  |  |  |  |  |  |  | t | **2.31** |  |  |  |  |  | **1.97** |  |
| Type of delivery | 434 |  | |  |  |  |  |  |  |  |  | p | **0.02** |  |  |  |  |  | **0.04** |  |
| Vaginal |  |  | |  |  |  |  |  |  |  |  |  |  |  | -0.02 | 0.99 |  |  | 0.04 | 1.02 |
|  |  |  | |  |  |  |  |  |  |  |  |  |  |  |  |  |  |  |  |  |
| C-section |  |  | |  |  |  |  |  |  |  |  |  |  |  | -0.27 | 0.67 |  |  | -0.16 | 0.73 |
|  |  |  | |  |  |  |  |  |  |  |  |  |  |  |  |  |  |  |  |  |
|  |  |  | |  |  |  |  |  |  |  |  | t |  |  | **2.64** |  |  |  | **2.06** |  |
|  |  |  | |  |  |  |  |  |  |  |  | p |  |  | **0.01** |  |  |  | **0.04** |  |
| Weight gain during pregnancy | 434 |  | |  |  |  |  |  |  |  |  |  |  |  |  |  |  |  |  |  |
| Exceeded |  |  | |  |  |  |  |  |  |  |  |  | 0.82 | 1.30 | 0.33 | 1.12 | 0.38 | 1.17 | 0.53 | 1.22 |
|  |  |  | |  |  |  |  |  |  |  |  |  |  |  |  |  |  |  |  |  |
| Non-exceeded |  |  | |  |  |  |  |  |  |  |  |  | 0.27 | 1.21 | -0.12 | 0.91 | 0.05 | 0.85 | -0.06 | 0.93 |
|  |  |  | |  |  |  |  |  |  |  |  |  |  |  |  |  |  |  |  |  |
|  |  |  | |  |  |  |  |  |  |  |  | t | **2.77** |  | **2.96** |  | **2.29** |  | **3.76** |  |
|  |  |  | |  |  |  |  |  |  |  |  | p | **0.01** |  | **<0.01** |  | **0.02** |  | **<0.001** |  |

Interactions between ACEs and polymorphisms of FTO and MC4R genes

|  | FTO |  | MC4R |  |
| --- | --- | --- | --- | --- |
| BMI z scores | F | p | F | p |
| ACE 0,1,2,3+ | **2.59** | **0.02** |  |  |
| Separation from parent | **3.06** | **0.048** | **3.07** | **0.03** |
| School problems | **5.73** | **0.004** |  |  |
| Other unspecified stressors | **4.60** | **0.01** |  |  |
| FMI z scores |  |  |  |  |
| ACE 0,1,2,3+ |  |  | **2.31** | **0.03** |
| Separation from parent |  |  | **8.34** | **<0.001** |
| School problems | **4.09** | **0.02** |  |  |
| Other unspecified stressors | **3.13** | **0.04** |  |  |
| FFMI z scores |  |  |  |  |
| Separation from parent | **3.27** | **0.04** |  |  |
| School problems | **4.95** | **0.007** |  |  |
| Other unspecified stressors | **3.03** | **0.04** |  |  |
| FatM z scores |  |  |  |  |
| ACE 0,1,2,3+ |  |  | **2.27** | **0.04** |
| Separation from parent |  |  | **9.73** | **<0.001** |
